# Integration of mRNA Expression Profile, Copy Number Alterations, and microRNA Expression Levels in Breast Cancer to Improve Grade Definition

**DOI:** 10.1371/journal.pone.0097681

**Published:** 2014-05-27

**Authors:** Claudia Cava, Gloria Bertoli, Marilena Ripamonti, Giancarlo Mauri, Italo Zoppis, Pasquale Anthony Della Rosa, Maria Carla Gilardi, Isabella Castiglioni

**Affiliations:** 1 Institute of Molecular Bioimaging and Physiology (IBFM), National Research Council (CNR), Milan, Italy; 2 Department of Informatics, Systems and Communications, University of Milan–Bicocca, Milan, Italy; Princess Margaret Cancer Centre, Canada

## Abstract

Defining the aggressiveness and growth rate of a malignant cell population is a key step in the clinical approach to treating tumor disease. The correct grading of breast cancer (BC) is a fundamental part in determining the appropriate treatment. Biological variables can make it difficult to elucidate the mechanisms underlying BC development. To identify potential markers that can be used for BC classification, we analyzed mRNAs expression profiles, gene copy numbers, microRNAs expression and their association with tumor grade in BC microarray-derived datasets. From mRNA expression results, we found that grade 2 BC is most likely a mixture of grade 1 and grade 3 that have been misclassified, being described by the gene signature of either grade 1 or grade 3. We assessed the potential of the new approach of integrating mRNA expression profile, copy number alterations, and microRNA expression levels to select a limited number of genomic BC biomarkers. The combination of mRNA profile analysis and copy number data with microRNA expression levels led to the identification of two gene signatures of 42 and 4 altered genes (FOXM1, KPNA4, H2AFV and DDX19A) respectively, the latter obtained through a meta-analytical procedure. The 42-based gene signature identifies 4 classes of up- or down-regulated microRNAs (17 microRNAs) and of their 17 target mRNA, and the 4-based genes signature identified 4 microRNAs (Hsa-miR-320d, Hsa-miR-139-5p, Hsa-miR-567 and Hsa-let-7c). These results are discussed from a biological point of view with respect to pathological features of BC. Our identified mRNAs and microRNAs were validated as prognostic factors of BC disease progression, and could potentially facilitate the implementation of assays for laboratory validation, due to their reduced number.

## Introduction

Breast cancer (BC) is a heterogeneous disease with varied morphological presentation, molecular features, behaviors, and response to therapy [1]–[2]. Clinical decisions on BC treatment are based on the availability of strong prognostic and predictive factors to guide the patient decision-making and the choice of treatment options [3]–[5]. One of the most well-established prognostic factors for BC is histological grade, which involves morphological assessment of tumor biological characteristics and quantifies tumor aggressiveness [6]–[7]. The histological definition of the tumor grade in BC is mainly based on the degree of differentiation of the tumor tissue [6]: grade 1 (G1) is a well-differentiated, slow-growing tumor; grade 3 (G3) is a poorly differentiated, highly proliferative tumor; grade 2 (G2) is a moderately differentiated, slightly faster-growing tumor than normal cells.

The prognostic value of histological grade has been documented for most tumor types [4]. The histological grade of BC has been correlated with life expectancy of patients [8]. For example, untreated patients with G1 disease have been shown to have a 95% 5-year survival rate, patients with G3 malignancies show 75% 5-year survival rates, whereas those with G2 malignancies show 50% 5-year survival rates [8]. For its excellent outcome G1 does not require adjuvant chemotherapy, on the contrary, G3 requires systemic treatment, while G2 is not useful for the treatment decision.

Mis-assignments of G1 to G3 grade or vice versa are rarely reported, while difficulties in discriminating G2 from the other grades are often presented [6]. In fact, a high percentage of tumors (30–60%) are classified as histologic G2 with poor degree of concordance between two different pathologists. Sometimes, a central pathologist consensus is used to improve pathology classification [9]–[10].

In recent years, molecular techniques, in particular gene expression profiling, have been used increasingly, in order to improve BC classification and to assess patient prognosis and response to therapy.

Most molecular studies of BC have focused on the analysis of only one or the combination of two genome-wide microarray-based expression profiling approaches, such as mRNA expression profiling, DNA copy number, and/or epigenetic analysis (e.g. microRNAs).

When only genome-wide microarray-based expression profiling was used, two different strategies were adopted to provide prognostic information by means of gene expression signatures [11]: following a “top-down” strategy, mRNA expression profiling from patients with known clinical outcome were statistically compared to identify signatures associated with different prognosis, without any biological assumption [12]; following a “bottom-up” strategy, mRNA expression profiling from patients with different tumor biological characteristics were selected and reduced in number following analysis through multivariate models [13]–[15], with a potential cost reduction of genomic biomarker analysis.

However, a different strategy, fully based on biological assumptions, implies the combination of two or more genome-wide microarray-based expression profiling resulting in the identification of molecular profiles able to predict cancer progression [16]–[19] and treatment response [16] but at the same time allowing the selection of only a limited number of target genes (e.g. from thousands to fifty) [20]–[21].

Most studies concerning DNA copy number alterations (CNAs) investigated the use of genetic aberrations as biomarkers for cancer prognosis [22]–[23], but few studies have been reported regarding the relationship between CNAs and disease progression [24]–[27]. Moreover, most of these studies did not consider that identifying CNAs in genes is important for defining key genetic events leading to malignant transformation and disease progression. The association between CNAs and gene expression levels has been demonstrated, and 12% of gene expression variation can be explained by differences in CNAs [28]. Genes responsible for regulating molecular processes may be targeted by these alterations, with expression changes resulting from CNAs. By combining gene expression and copy number data, genes involved in tumor processes can be better characterized and numerically reduced.

Only a limited number of studies have used this approach in cancer prognosis [29]–[44]. Several studies have used high-resolution oligonucleotide comparative genomic hybridization arrays, and, by matching gene expression array data, they demonstrated a correlation between DNA copy number alteration and mRNA levels [29]–[31]. Chin et al. [37] showed that the accuracy of risk stratification, according to the outcome of BC, could be improved through a combined analysis of gene expression and DNA copy number. Other studies [36]–[41] have correlated DNA copy number changes with gene expression signatures.

MicroRNAs (miRNAs) are small, noncoding RNA molecules approximately 22 nucleotides in length that interact with their target mRNAs to inhibit translation or target mRNA for degradation or deadenylation [45]–[46]. This interaction is guided by sequence complementarity and results in the reduction of mRNA, causing decreasing of protein levels.

Each miRNA is potentially able to regulate approximately 100 or more mRNA targets, and 30% of all human genes are thought to be regulated by miRNAs [47]–[49]. miRNAs are involved in key biological processes, such as development, differentiation, apoptosis, and proliferation [50]–[51]; therefore, identification and validation of miRNA–mRNA target interactions is essential. miRNA expression is highly specific for tissues and developmental stages [51]–[52], and has recently been used for the molecular classification of tumors [53]–[54]. Zhang et al. [55] showed that CNAs of miRNAs and their regulatory genes is highly prevalent in cancer.

In addition to deregulated expression of miRNAs associated with a variety of cancers [56]–[57], in 2008 it was discovered that miRNAs are also present in blood of cancer patients [58]–[60]. Over the last few years, these results were confirmed in other cancer studies and in different diseases, (for a review see [61]–[64]), and circulating miRNAs have emerged as promising novel and minimally invasive markers [65]. Circulating, cell-free miRNAs hold great promise as a new class of biomarkers [66]–[69] due to their surprisingly high stability in plasma, association with disease states, small amounts of starting materials needed and ease of sensitive measurement. MicroRNA blood profiles may be useful to classify different types of cancer, and also may be considered as potential targets to be obtained in blood in an early stage of disease or hopefully when the disease is not expressed yet [70]–[71], and also to discriminate between benign and malignant disease [71].

In a limited number of studies involving integration analysis of mRNA expression in BC, genomic changes and miRNA expression were adopted [72]–[75]. Eo et al. [72] classified BC subtypes to incorporate pathways information with various genetic analyses and achieved better performance than classifiers based on the expression levels of individual genes of BluePrint. Kristensen et al. [74] used an integrated approach to identify and classify BC according to the most deregulated pathways that provide the best predictive value with respect to prognosis, as well as identified key molecular and stromal signatures.

By combining the analysis of mRNA expression data, array-comparative genomic hybridization (aCGH), and miRNAs, Blenkiron et al. [75] identified a number of miRNAs that are differentially expressed among molecular tumor subtypes.

The aim of this study was to develop a method able to efficiently combine CNA, miRNAs and mRNAs in order to reclassify histological G2 BC tumor into G1- and G3-like BC tumor, thus improving BC grade definition. Our fully biological-based approach is novel with respect to previously published approaches proposed for similar purposes based on only mRNA expression profiling [14]–[15], and considers the combined effect of epigenetic and genetic changes resulting in deregulated gene expression and function.

We also assessed if the proposed combined approach allows incremental results in BC classification with respect to those previously obtained in published papers [14]–[15], in terms of both grade classification performance and number and type of genomic features identified as candidate biomarkers of BC progression and potentially suitable for an easy and less expensive implementation of clinical assays.

The identified CNA-altered mRNA-targets and miRNA could be further investigated in laboratory by clinical experiments on tissue or blood samples from BC patients, as potential prognostic biomarkers responsible of BC disease development and progression, thus resulting very useful for treatment decision.

## Materials and Methods

### Gene expression analysis: mRNAs

We used 3 public BC microarray datasets from the Gene Expression Omnibus (GEO) database: the dataset used by Foekens et al. (FK) [76], GSE11121, and GSE2990 containing 180, 200, and 125 samples, respectively, for a total of 505 BC microarray data sets. Datasets were all from the same Affymetrix GeneChip Human Genome U133A platform. These data sets were subjected to two phases:

- Normalization. Gene expression values were computed from microarray data using a robust multi-array average (RMA) method.
- Data merging. To harmonize gene expression data from the three different datasets, it was necessary to detect and remove the batch effects (experimental variations of datasets generated by different laboratories). An empirical Bayes method, combining batches of gene expression microarray data (ComBat), was used. The systematic difference for differently normalized data generated by the three laboratories was adjusted [77]–[78].

From the 505 BC microarray data sets, properly normalized and harmonized, we randomly selected three groups: microarray data sets from 78 patients with G1 BC, microarray data sets from 78 patients with G2 BC and microarray datasets from 78 patients with G3 BC. This selection was performed in order to use equal sample sizes from the three groups of microarray datasets.

To identify associations between gene expression and disease progression with regard to grade, a significance analysis of microarray (SAM) was used [79]. SAM was applied to select statistically significant genes based on differential expression between 2 classes of samples. SAM identifies statistically significant genes by carrying out a gene-specific *t*-test with respect to the separation of the 2 classes of interest, and then computes a statistic measure for each gene which represents the strength of the relationship between gene expression and a response variable (e.g. false discovery rate, FDR). More specifically, as a first step, SAM analysis was used to detect DNA probes to discriminate between the 2 following classes of interest: G1 vs G3, G1 vs G2 and G2 vs G3.

The genes were considered up- or down-regulated if their mean expression in one class were significantly higher/lower respectively (FDR, q-value <0.01) than in the other class. In a second step, the up- or down- regulated genes were identified by submitting the corresponding Ids probes from the HGU133 Array to Affymetrix through the Netaffx tool [80].

We compared deregulated genes obtained from G1 vs G2 SAM analysis and from G2 vs G3 SAM analysis with deregulated genes obtained from G1 vs G3 SAM analysis.

### Copy number alteration analysis: CNA-associated mRNAs

We used one public BC SNP array dataset from the GEO database: GSE16619. We selected 9 patients with G1 and 66 patients with G3 BC. All samples were characterized using the Affymetrix SNP 5.0 array. We used the copy number analyzer for GeneChip (CNAG) [81] to identify the chromosomal regions with gains and losses of DNA and we used the UCSC table browser [82] to identify genes within the identified chromosomal regions.

In both G1 and G3 groups, we selected the first 6000 more frequently observed genes with CNA.

### miRNA analysis: miRNA-regulated mRNAs

We used one public BC miRNAs expression data set from the GEO database: GSE22216. We selected 42 patients with G1 and 42 patients with G3 BC.

**Figure 1:**
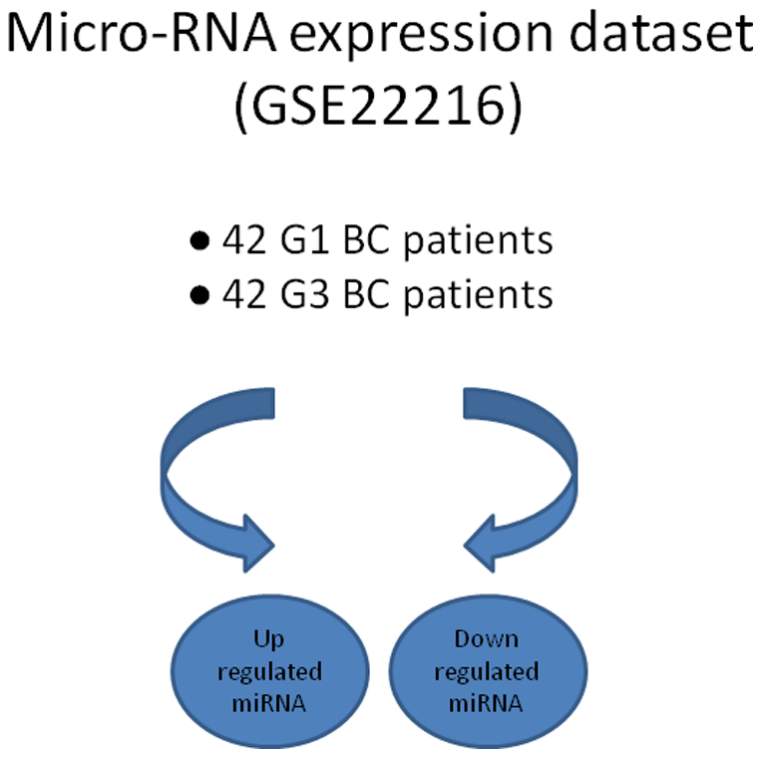
Schematic procedure for the identification of up and down regulated miRNAs. Identification of up and down regulated miRNAs.

SAM was applied to select significant miRNAs based on differential expression between these two classes of samples (Fig. 1). miRNA were considered up- or down-regulated if their mean expression in G3 BC were significantly higher or lower, respectively (FDR, q-value <0.01) than in class G1 BC.

Each miRNA can regulate approximately 100 or more mRNA targets [48]. miRDB [83]–[84] was used to identify mRNA targets of each miRNA obtained in the differential expression analysis.

### Combination of gene expression and genome copy number alteration

**Figure 2:**
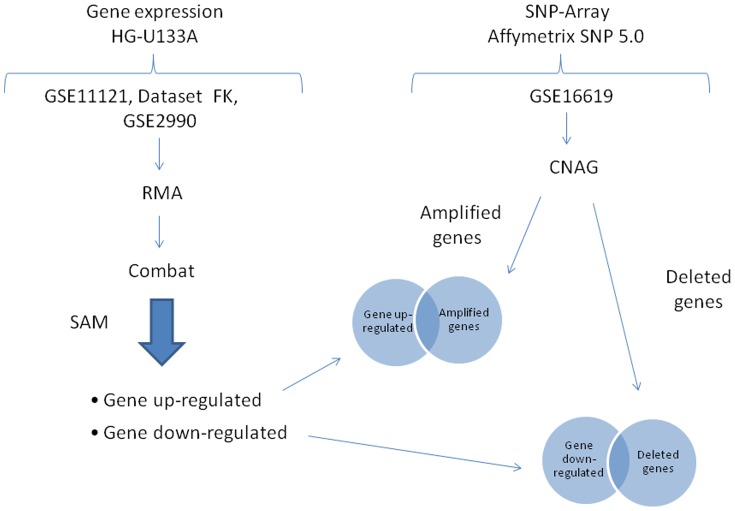
Schematic procedure Gene Expression and Genome CNA. Identification of up regulated genes with copy number gains and down regulated genes with copy number losses is showed.

In this phase, identification of differentially expressed genes with CNAs (gains/losses) was obtained (Fig. 2). In particular, by considering the results of gene expression analysis (i.e. up- and down-regulated genes) and of copy number analysis (i.e. amplified and deleted genes), we selected the following genes:

- Up-regulated genes with copy number gains in G1 BC patients (by selecting genes common to the set of up-regulated and the set of amplified genes);
- Down-regulated genes with copy number losses in G1 BC patients (by selecting genes common to the set of down-regulated and the set of deleted genes);
- Up-regulated genes with copy number gains in G3 BC patients (by selecting genes common to the set of up-regulated and the set of amplified genes);
- Down-regulated genes with copy number losses in G3 BC patients (by selecting genes common to the set of down-regulated and the set of deleted genes);

### Combination of gene expression, genome copy number alteration and miRNA-analysis

**Figure 3:**
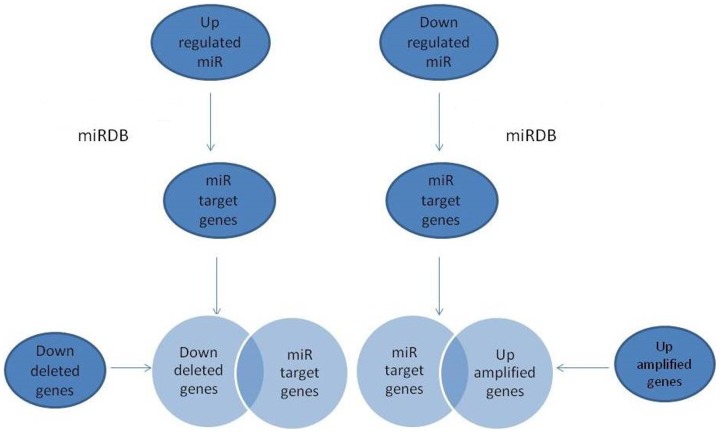
Schematic procedure miRNAs analysis. Combination of gene expression, genome CNA, and miRNA-regulated mRNAs.

We hypothesized that if a miRNA is up-regulated in cancer, it down-regulates a gene that can act as a tumor suppressor or transcriptional repressor of an oncogene. In contrast, if a miRNA is down-regulated in cancer, its target gene is up-regulated, which can be an oncogene or a transcriptional repressor of an oncosuppressor. Even if CNA revealed mRNA deregulation, the combination of miRNAs and CNA on the genes may reveal other mRNA deregulation. We analyzed the target genes of up- and down-regulated miRNAs from G1 and G3 BC patients. These target genes were compared with up-regulated and amplified (up-amplified) genes and down-regulated and deleted (down-deleted) genes, respectively. We then selected common genes to the set of i) down-regulated and deleted genes, with their up-regulated miRNAs-mRNA and ii) up-regulated and amplified genes, with their down-regulated miRNAs-mRNA (Fig. 3).

In particular, we identified:

- Up-regulated genes with copy number gains that are targets of down-regulated miRNAs in patients with G1 BC;
- Down-regulated genes with copy number losses that are targets of up-regulated miRNAs in patients with G1 BC;
- Up-regulated genes with copy number gains that are targets of down-regulated miRNAs in patients with G3 BC;
- Down-regulated genes with copy number losses that are targets of up-regulated miRNAs in patients with G3 BC.

### The classifier

We designed a machine learning algorithm, a rapid miner workflow (RMA-WF) [43]–[44] [85]–[87] based on support vector machines (SVM).

RMA-WF was used for the validation of the classifier following two different processes.

– Cross-validation operator. We used a *k*-fold cross validation process in which a two-step process was performed. In the first step, the classifier was trained over a predetermined set of G1 and G3 BC data. In the second step, the trained classifier was used to test new classification exemplars. Specifically, the G1 and G3 BC datasets were partitioned into *k* subsets of equal size. Of the *k* subsets, a single subset was retained as the testing data set and the remaining *k−1* subsets were used as the training data set. The cross-validation process was then repeated *k* times, with each of the *k* subsets used exactly once as the testing data. The *k* results from the *k* iterations were averaged to produce a single estimation. Mean accuracy, sensitivity, and specificity of the classifier were determined. It is worth noting that, in this work, specificity relates to the ability of the classifier to identify G1 samples (as it measures the percentage of G1 samples which are correctly identified as belonging to G1 class), and sensitivity relates to the ability of the classifier to identify G3 samples (as it measures the percentage of G3 samples which are correctly identified as belonging to G3 class).

Area under the receiver operating characteristic curve (AUC) was also computed as a measure of classifier performance.

-Training and testing validation operator. In the first step, the classifier was trained over a predetermined set of G1 and G3 BC data. In the second step, the trained classifier was used to test new classification examples of G2 data, which were re-classified as G1 or G3 (G1*, G3*) after testing. In a third step, these classes G1* and G3* were used to train again RMA-WF and to test G1 and G3 datasets.

Mean accuracy, sensitivity, specificity, AUC and computational time of the classifier were determined.

We optimized inference accuracy over a space of given SVM feasible learning parameters: kernel.γ, kernel.C ∈{ 0…5} step 30; kernel.type  =  RADIAL, DOT, ANOVA (see Rapid Miner documentation at [85]). This approach allowed to find, the best SVM learning parameters for each data type over the same space of values.

All data have been deposited in our research centre repository (inlab.ibfm.cnr.it/research_data.php).

### Evaluation of combination approaches

The performances of BC grade classification was evaluated using the genomic biomarkers selected by the different combination approaches:

- I: The expression levels of the up- or down-regulated mRNAs as obtained from gene expression analysis;
- II: The expression levels of the up-regulated genes with amplification and down-regulated genes with deletion and the expression levels as obtained from the combined analysis of gene expression and genome CNA;
- III: The expression levels of i) up-regulated and copy number-amplified genes (up-amplified) that are target of down-miRNAs and ii) down-regulated and copy number-deleted genes (down-deleted) that are target of up-miRNAs, as obtained from the combined analysis of gene expression, genome CNA, and miRNA.
- IV: The expression levels of miRNAs, as obtained from the combined analysis of gene expression and genome CAN

### Evaluation of G1 vs. G3 classification

The classifier was tested to distinguish between histological G1 and G3 BC patients using the different combination approaches (I–IV).

Cross validation of the classifier was performed for 5 different BC datasets: 17 G1 BC patients and 17 G3 BC patients from the FK dataset, 29 G1 BC patients and 29 G3 BC patients from the GSE11121 dataset, 28 G1 BC patients and 28 G3 BC patients from the GSE2990 dataset, 30 G1 BC patients and 30 G3 BC patients from the GSE7390 dataset, and 42 G1 BC patients and 42 G3 BC patients from the GSE22216 (miRNA- dataset). The value *k* was adjusted with k = 10 as in references [88]–[90]. Confidence intervals were stated at the 95% confidence level.

### Evaluation of G2 classification

The classifier was tested to re-classify G2 using the different combination approaches (I–IV).

For this purpose, the machine learning algorithm based on SVM (RMA-WF) was used to classify G2 BC patients in G1 or G3 class. Specifically, 5 different BC datasets were used: from the FK dataset, 17 G1 BC patients and 17 G3 BC patients were used for training and 34 G2 patients were used for testing; from the GSE11121 dataset, 29 G1 BC patients and 29 G3 BC patients were used for training and 50 G2 patients were used for testing; from the GSE2990 dataset, 28 G1 BC patients and 28 G3 BC patients dataset were used for training and 44 G2 patients were used for testing; from the GSE7390 dataset 30 G1 BC patients and 30 G3 BC patients were used for training and 34 G2 patients were used for testing. From the miRNA dataset GSE22216, 42 G1 BC patients and 63 G3 BC patients were used for training and 74 G2 patients were used for testing.

As results of testing, G2 samples were classified as G1 or G3 (G1*, G3*). These classes G1* and G3* were used to train again RMA-WF and to test to the 5 different G1 and G3 BC datasets.

### Evaluation of our gene signature in comparison with other gene signatures

Our gene signature (III) was compared with Sotiriou et al. 97-gene signature [14], Ivshina et al. 18-gene signature [15] and Ivshina et al. 6-gene signature [15], obtained by previous studies on datesets of mRNA expression profiling of BC patients with the same purpose of improving grade definition.

From above comparisons, we obtained a downsized gene signature consisting of genes, which were shared with the above-mentioned signatures. This downsized gene signature was considered as a new gene signature (V).

Our final gene signatures (III and V) were tested together with Sotiriou et al. 97-gene signature, Ivshina et al. 18-gene signature, and Ivshina et al. 6-gene signature, in terms of both grade classification performance and prognostic value. In order to avoid cohort-specific biases, we used BC datasets not employed in any of the above-referenced studies in the process of gene signature identification.

### Evaluation of G1 vs. G3 classification

The classifier was tested to distinguish between histological G1 and G3 BC patients using the five above mentioned gene signatures. The ability of the classifier to distinguish between G1 and G3 BC patients was evaluated with a cross-correlation approach: 30 G1 and 30 G3 BC patients from the GSE7390 dataset were used, and 28 G1 BC patients and 28 G3 BC patients from the Stockholm dataset [91].

### Evaluation of G2 classification

The classifier was tested to re-classify G2 using the five gene signatures. For this purpose, the classifier was trained to the 2 different BC datasets used previously: from the GSE7390 dataset, 30 G1 BC patients and 30 G3 BC patients were used for training and 34 G2 patients were used for testing; from the Stockholm dataset (GSE1456), 28 G1 BC patients and 28 G3 BC patients were used for training and 38 G2 patients were used for testing.

A survival analysis was also performed, using the survival package included in the *R* statistical analysis software [92]–[93].

To determine if the re-classification of G2 in G1* and G3*correlates with patient survival endpoints, we examined relapse-free survival of patients. The Kaplan-Meier estimate was used to compute survival curves. Log-Rank tests and hazard ratios [C.I. 95%] were computed to assess the statistical significance of the differences between G1*and G3*, G1 and G1*, G3 and G3*.

## Results

### Differential gene expression analysis

The gene expression analysis of G1 vs G3 BC samples allowed identification of 1190 de-regulated genes (1392 probes). Among these, 578 (687 probes) were found to be up-regulated in G3 and 612 (705 probes) were found to be down-regulated in G3. The functions of the 578 up-regulated genes have been previously associated with cell cycle control, mitosis and mitotic spindle regulation or DNA repair. 66/578 genes overlap with the 66/80 G3-upregulated genes described by Sotiriou et al. [14] (i.e. BIRC5, CCNA2, FOXM1, KPNA2, MYBL2, TPX2, UBE2N/2S); moreover, 10/578 genes overlap with the 10/16 G3-upregulated genes reported in Ivshina A. et al [15]. About the remaining 502 up-regulated genes, their main functions are linked to cell cycle regulation (i.e. PLK1, PLK4, …); protein folding (i.e. DNAJA2, HSPD1,…); DNA (i.e. CDT1) and RNAs (i.e. DKC1, EIF2C2…) maturation; DNA repair (i.e. EXO1, EXOSC2,…) and replication control (i.e. GINS1, MCM2, MCM10, NCAPD2, NCAPG,…); genome stability (spindle control, i.e. KIF family; nucleosome control, i.e. HMG family). About the 612 G3-downregulated genes, the main pathways affected are apoptosis (i.e. BCL2, CASP9), cell cycle control (i.e. CDKN1C, CREBL2, DUSP1,…), transcription regulation (i.e. CTDSP1, CTDSPL, DDX17,…), cell adhesion (i.e.ADAM12, ATP7A, CD134, CD302), but also remodeling of cytoskeleton (i.e.KIF13B, LIMA1, LAMA2, LAMB2, LAMC1…) and external matrix (i.e. collagen components as COL14A1, COL16A1,…).

The gene expression analysis of G1 vs G2 BC samples allowed identification of 40 de-regulated genes (40 probes). Among these, 36 (36 probes) were found to be up-regulated in G2 BC and 4 (4 probes) were found to be down-regulated in G2 BC.

The gene expression analysis of G2 vs G3 BC samples allowed identification of 160 de-regulated genes (171 probes). Among these, 127 (138 probes) were found to be up-regulated in G3 BC and 33 (33 probes) were found to be down-regulated in G3 BC.

From the comparison of de-regulated genes obtained from G1 vs G2 SAM analysis and from G2 vs G3 SAM analysis with de-regulated genes obtained from G1 vs G3 SAM analysis, we found:

- between G1 vs G2 and G1 vs G3 no gene specifically associated with G2 BC. All 4 down-regulated genes in G2 (G1 vs G2) were found common to 4/612 up-regulated genes in G1 patients (G1 vs G3). Similarly, all 36 up-regulated genes in G2 (G1 vs G2) were found common to 36/578 down-regulated genes in G1 BC patients (G1 vs G3).
- between G2 vs G3 and G1 vs G3 few genes associated with G2 BC. 124/127 (98%) down-regulated genes in G2 (G2 vs G3) are common to 124/578 down-regulated genes in G1 BC patients (G1 vs G3). 32/33 (97%) up-regulated genes in G2 (G2 vs G3) are common to 32/612 up-regulated genes in G1 BC patients (G1 vs G3).

Our results show that G2 BC is most likely a mixture of G1 and G3 BC that have been somehow misclassified, as previously reported [14]–[15].

### Copy number alteration analysis

Copy number gains were frequently observed within chromosomes 1q, 8q, 17q, and 20; copy number losses were frequently observed within chromosomes 13q, 1p, and 3. Our findings were consistent with results of previous cytogenetic studies [28] [94].

**Figure 4:**
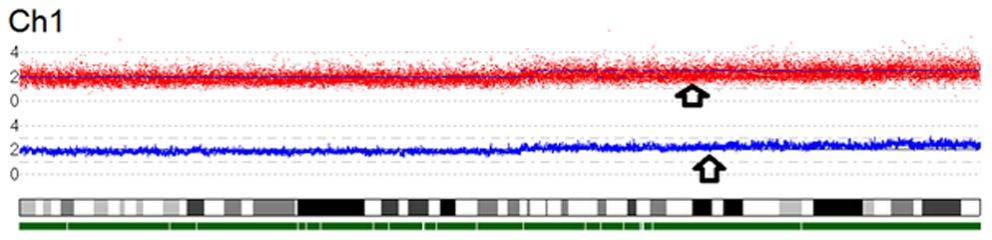
Example of CNA detected by Genome Wide 5.0 Picture generated with CNAG. The red plots (top panel) show individual SNP probe signals. In the second panel the blue line represents total gene dosage. The diploid state is indicated by 2. The chromosome 1 shows one big amplification in 1q.

Fig. 4 shows an example of CNA detected in a Genome Wide 5.0 picture generated with CNAG, where chromosome 1 shows one large amplification in 1q.

The average number of deleted regions was 29 in G1 patients and 39 in G3 patients. The average number of amplified regions was 97 in G1 patients and 179 in G3 patients.

The average number of deleted genes was 645 in G1 patients and 266 in G3 patients. The average number of amplified genes was 573 in G1 patients and 331 in G3.

### miRNA analysis

miRNA analysis of G1 vs G3 BC samples allowed to identify 26 up-miRNAs and 53 down-miRNAs.

### miRNA-regulated mRNAs

mRNA targets of each miRNA were identified in the differential expression analysis:

- The average number of target genes (total target genes/miRNAs) were 41 from up-regulated miRNAs and 44 from down-regulated miRNAs;

### Combination of gene expression and genome CNA

Up- and down-regulated genes with CNAs were selected. Specifically, the following genes were selected:

- 108 up-regulated genes with copy number gains were found in G1 BC patients.
- 123 down-regulated genes with copy number losses were found in G1 BC patients.
- 151 up-regulated genes with copy number gains were found in G3 BC patients.
- 150 down-regulated genes with copy number losses in G3 BC patients.

### Combination of gene expression, genome CNA, and miRNA-regulated mRNAs

We identified the following classes:

- 3 down-regulated miRNAs that target up-amplified genes reported previously in G1 BC patients;
- 8 up-regulated miRNAs that target down-deleted genes reported previously in G1 BC patients;
- 8 down-regulated miRNAs that target up-amplified genes reported previously in G3 BC patients.
- 7 up-regulated miRNAs that target down-deleted genes reported previously in G3 BC patients.

**Figure 5:**
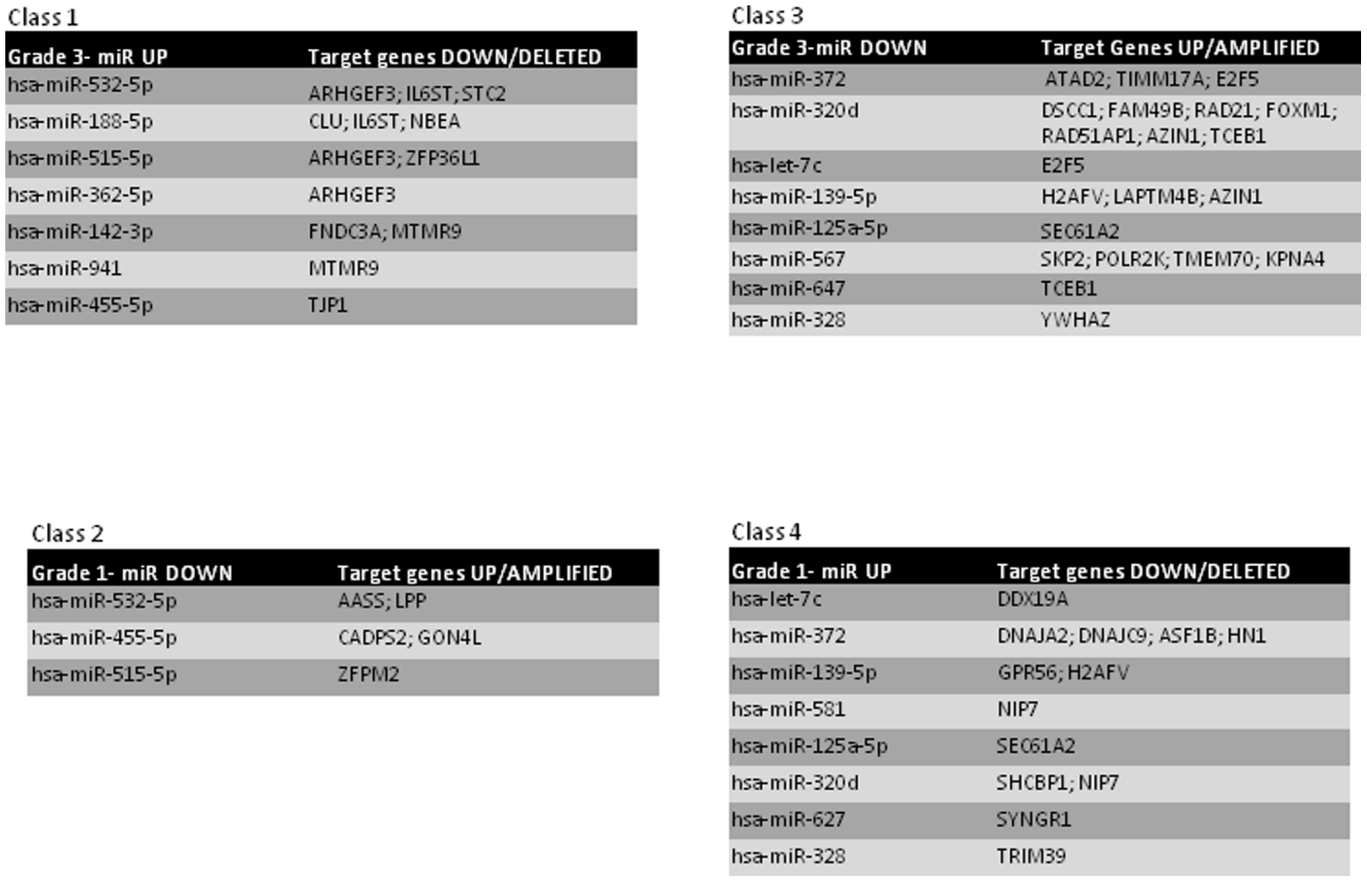
Combination of Gene Expression - Genome CNA and miRNA analysis. Each table represents: down-regulated genes with putative up-regulated miRNAs in G3 (Class 1), up-regulated genes with putative down miRNAs in G1 (Class 2), up-regulated genes with putative down-regulated miRNAs in G3 (Class 3), and down-regulated genes with putative up miRNAs in G1 (Class 4), respectively.

In particular, we found the following genes, as shown in Fig. 5:

- 13 down-regulated genes, with copy number losses, that are targets of up-regulated miRNAs in patients with G3 BC (Class 1)
- 5 up-regulated genes, with copy number gains, that are targets of down-regulated miRNAs in patients with G1 BC (Class 2)
- 21 up-regulated genes, with copy number gains, that are targets of down-regulated miRNAs in patients with G3 BC (Class 3)
- 13 down-regulated genes, with copy number losses, that are targets of up-regulated miRNAs in patients with G1 BC (Class 4)

### Evaluation of G1 and G3 classification

For each combined approach (I: gene expression, II: combination of gene expression, and genome CNA, III: combination of gene expression - genome CNA, and miRNA analysis, IV: miRNAs), the results of G1 vs G3 BC classification are presented in Tables 1–4 for accuracy, sensitivity, specificity and AUC, respectively.

**Table 1:** G1 vs. G3 classification performances (mRNA and miRNA): accuracy, cross-validation.

ACCURACY				
	I 1390 genes	II 532 genes	III 42 genes	IV 17 miRNA
FK 17G1vs17G3	82.50% [CI 95%] 77.57–87.42	80.00% [CI 95%] 75.44–84.55	84.17% [CI 95%] 76.81–91.52	
Gse11121 29G1vs29G3	90.00% [CI 95%] 87.15–92.84	91.67% [CI 95%] 88.21–95.12	93.00% [CI 95%] 90.78–95.21	
GSE2990 28G1vs28G3	93.33% [CI 95%] 90.43–96.22	93.33% [CI 95%] 90.43–96.22	95.00% [CI 95%] 92.99–97	
GSE7390 30G1vs30G3	90.00% [CI 95%] 86.62–93.37	90.00% [CI 95%] 87.20–92.79	90.00% [CI 95%] 87.2–92.79	
GSE22216 42G1vs42G3				85.69% [CI 95%] 84.12–87.25
**Mean (over datasets)**	**88.95%+/−4.58** 1	**88.75%+/−5.98** 1	**90.54%+/−4.71** 1	**85.69%**

1 standard deviation.

**Table 2:** G1 vs. G3 classification performances (mRNA and miRNA): sensitivity, cross-validation.

SENSITIVITY				
	I 1390 genes	II 532 genes	III 42 genes	IV 17 miRNA
FK 17G1vs17G3	90.00% [CI 95%] 83.27–96.72	85.00% [CI 95%] 74.23–95.76	75.00% [CI 95%] 61.45–88.54	
Gse11121 29G1vs29G3	90.83% [CI 95%] 87.18–94.47	90.00% [CI 95%] 84.5–95.49	90.00% [CI 95%] 86.06–93.93	
GSE2990 28G1vs28G3	97.50% [CI 95%] 95.53–99.46	96.67% [CI 95%] 94.05–99.28	96.67% [CI 95%] 94.05–99.28	
GSE7390 30G1vs30G3	93.33% [CI 95%] 89.95–96.7	96.67% [CI 95%] 94.13–99.2	100.00% [CI 95%] 100–100	
GSE22216 42G1vs42G3				86.83% [CI 95%] 82.07–91.58
**Mean (over datasets)**	**92.91%+/−3.36** 1	**92.08%+/−5.6** 1	**90.41%+/−11.08** 1	**86.83%**

1 standard deviation.

**Table 3:** G1 vs. G3 classification performances (mRNA and miRNA): specificity, cross-validation.

SPECIFICITY				
	I 1390 genes	II 532 genes	III 42 genes	IV 17 miRNA
FK 17G1vs17G3	76.67% [CI 95%] 65.69–87.64	65.00% [CI 95%] 51.87–78.12	96.67% [CI 95%] 93.3–100	
Gse11121 29G1vs29G3	90.00% [CI 95%] 86.06–93.93	93.33% [CI 95%] 89.89–96.76	96.67% [CI 95%] 94.09–99.24	
GSE2990 28G1vs28G3	88.33% [CI 95%] 83.52–93.13	90.00% [CI 95%] 84.41–95.58	91.67% [CI 95%] 87.19–96.14	
GSE7390 30G1vs30G3	86.67% [CI 95%] 81.07–92.26	83.33% [CI 95%] 77.67–88.98	80.00% [CI 95%] 74.4–85.59	
GSE22216 42G1vs42G3				83% [CI 95%] 79.61–86.38
**Mean (over datasets)**	**85.41%+/−5.98** 1	**82.91%+/−12.64** 1	**91.25%+/−7.86** 1	**83%**

1 standard deviation.

**Table 4:** G1 vs G3 classification performance (mRNA and miRNA): AUC, cross-validation.

AUC				
	I 1390 genes	II 532 genes	III 42 genes	IV 17 miRNA
FK 17G1vs17G3	0.767 [CI 95%] 0.65–0.87	0.733 [CI 95%] 0.62–0.84	0.850 [CI 95%] 0.74–0.95	
Gse11121 29G1vs29G3	0.967 [CI 95%] 0.94–0.99	0.956 [CI 95%] 0.93–0.97	0.967 [CI 95%] 0.95–0.98	
GSE2990 28G1vs28G3	0.964 [CI 95%] 0.94–0.98	0.978 [CI 95%] 0.96–0.99	0.940 [CI 95%] 0.91–0.96	
GSE7390 30G1vs30G3	0.956 [CI 95%] 0.93–0.97	0.933 [CI 95%] 0.90–0.96	0.900 [CI 95%] 0.86–0.93	
GSE22216 42G1vs42G3				0.87 [CI 95%] 0.84–0.89
**Mean (over datasets)**	**0.91+/−0.09** 1	**0.9+/−0.11** 1	**0.91+/−0.05** 1	**0.87**

1 standard deviation.

Although the combination strategy allowed to reduce the number of genes from 1390 to 42, all mRNA signatures derived by the three approaches (I, II, III) achieved good and similar mean performance. The combination of gene expression, genome CNA, and miRNA-regulated mRNAs (III) slightly improved mean accuracy and sensitivity with respect to I and II approaches, achieving mean values >90% (mean accuracy: 90.54% vs 88.95% and 88.75%, mean specificity: 91.25% vs 85.41% and 82.91%;). Mean sensitivity was slightly worsen, however >90% (90.41% vs 92.91% and 92.08%).

AUC results (Table 4) showed the good and similar performances of the classifier when all the proposed approach I, II, III were applied.

The combination strategies allowed to identify 17 miRNA representing an epigenetic signature able to achieve good performance in G1 vs G3 classification (>80% for all indexes), although lower than the mRNA-based approaches (I, II, III).

Table 5 shows the computational times required by the classification algorithm for the different proposed approaches (Computer processor: Intel Core i5-3330S CPU @ 2.70 GHz), showing the improvement in the computation performances with the biomarker number reduction.

**Table 5:** Classification performances (mRNA and miRNA): computational time (sec, execution time/number of samples), cross-validation on G1–G3.

EXECUTION TIME (sec)				
	I 1390 genes	II 532 genes	III 42 genes	IV 17 miRNA
FK 17G1vs17G3	43.32	18.6	2.2	
Gse11121 29G1vs29G3	53.48	23.9	2.6	
GSE2990 28G1vs28G3	51.67	22.9	2.7	
GSE7390 30G1vs30G3	57	25.8	3.2	
GSE22216 42G1vs42G3				4.7

### Evaluation of G2 classification

For each combined approach (I: gene expression, II: combination of gene expression, and genome CNA, III: combination of gene expression - genome CNA, and miRNA analysis, IV: miRNA), the results of G1 vs G3 BC classification patients starting from the re-classified G2 BC patients (in G1* and G3*), are presented in Tables 6–9 for accuracy, sensitivity, specificity and AUC, respectively.

**Table 6:** Classification performances (mRNA and miRNA): accuracy, TRAINING on G1*-G3*, TESTING on G1–G3.

ACCURACY				
	I 1390 genes	II 532 genes	III 42 genes	IV 17 miRNA
FK 17G1*vs17G3*	79.41%	76.47%	76.47%	
Gse11121 25G1*vs25G3*	89.65%	87.93%	84.48%	
GSE2990 22G1*vs22G3*	87.5%	85.71%	91.07%	
GSE7390 17G1*vs17G3*	85%	91.66%	85%	
GSE22216 37G1*vs37G3*				82.85%
**Mean (over datasets)**	**85.39%+/−4.41** 1	**85.44%+/−6.46** 1	**84.25%+/−5.99** 1	**82.85%**

1 standard deviation.

**Table 7:** Classification performances (mRNA and miRNA): sensitivity,TRAINING on G1*–G3*, TESTING on G1–G3.

SENSITIVITY				
	I 1390 genes	II 532 genes	III 42 genes	IV 17 miRNA
FK 17G1*vs17G3*	76.74%	82.35%	70.58%	
Gse11121 25G1*vs25G3*	89.65%	86.2%	82.75%	
GSE2990 22G1*vs22G3*	85.71%	82.14%	89.28%	
GSE7390 17G1*vs17G3*	90%	100%	93.33%	
GSE22216 37G1*vs37G3*				88.88%
**Mean (over datasets)**	**85.52%+/−6.21** 1	**87.67%+/−8.42** 1	**83.98%+/−9.94** 1	**88.88%**

1 standard deviation.

**Table 8:** Classification performances (mRNA and miRNA): specificity, TRAINING on G1*–G3*, TESTING on G1–G3.

SPECIFICITY				
	I 1390 genes	II 532 genes	III 42 genes	IV 17 miRNA
FK 17G1*vs17G3*	82.35%	70.58%	82.35%	
Gse11121 25G1*vs25G3*	89.65%	89.65%	86.20%	
GSE2990 22G1*vs22G3*	89.28%	89.28%	92.85%	
GSE7390 17G1*vs17G3*	80%	83.33%	76.66%	
GSE22216 37G1*vs37G3*				73.80%
**Mean (over datasets)**	**85.32%+/−4.88** 1	**83.21%+/−8.9** 1	**84.51%+/−6.79** 1	**73.80%**

1 standard deviation.

**Table 9:** Classification performance (mRNA and miRNA): AUC, TRAINING on G1*–G3P, TESTING on G1–G3.

AUC				
	I 1390 genes	II 532 genes	III 42 genes	IV 17 miRNA
FK 17G1*vs17G3*	0.88	0.87	0.88	
Gse11121 25G1*vs25G3*	0.95	0.95	0.94	
GSE2990 22G1*vs22G3*	0.95	0.95	0.95	
GSE7390 17G1*vs17G3*	0.94	0.93	0.89	
GSE22216 37G1*vs37G3*				0.87
**Mean (over datasets)**	**0.93+/−0.03** 1	**0.92+/−0.03** 1	**0.91+/−0.03** 1	**0.87**

1 standard deviation.

All indexes showed good and similar mean performance of the classifier when the proposed approach I, II, III were applied. Consistently with our previous results (see Section “Evaluation of combination approach”), method IV (miRNA) achieved good performance for all indexes, although lower than the three mRNA-based approaches (I, II, III).

Table 10 shows computational time of classification algorithm for the different proposed approaches (Computer processor: Intel Core i5-3330S CPU @ 2.70GHz), confirming the improvement in the computation performances with the biomarker number reduction.

**Table 10:** Classification performances: computational time (second) Mean per sample (execution time/number of samples).

EXECUTION TIME				
	I 1390 genes	II 532 genes	III 42 genes	IV 17 miRNA
FK 17G1*vs17G3*	37.35	6.08	4.6	
Gse11121 25G1*vs25G3*	47.42	9.08	6.97	
GSE2990 22G1*vs22G3*	49.03	11.27	6.91	
GSE7390 17G1*vs17G3*	50.08	12.10	7.3	
GSE22216 37G1*vs37G3*				4.8

Intel Core i5-3330S CPU @ 2.70 GHz TRAINING-TEST.

**Figure 6:**
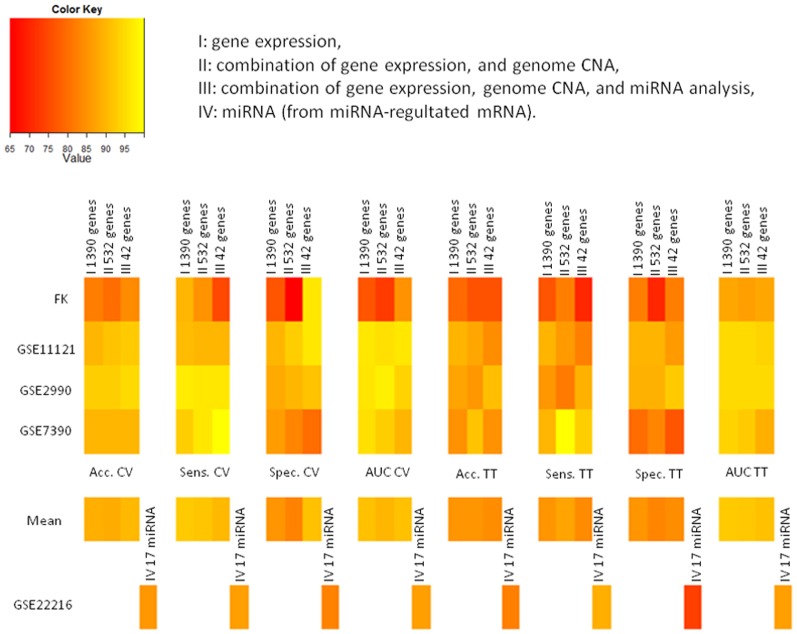
Heat maps of classification performances for each combined approach. I: gene expression, II: combination of gene expression, and genome CNA, III: combination of gene expression, genome CNA, and miRNA analysis, IV: miRNA (from miRNA-regultated mRNA). Classification performances were showed for: cross-validation (CV), and training on G1*–G3* - testing on G1–G3 (TT).

Figure 6 shows heat maps of classification performances for each combined approach (I,II,III,IV) for cross- validation and training on G1*–G3*, testing on G1–G3 (TT).

**Figure 7:**
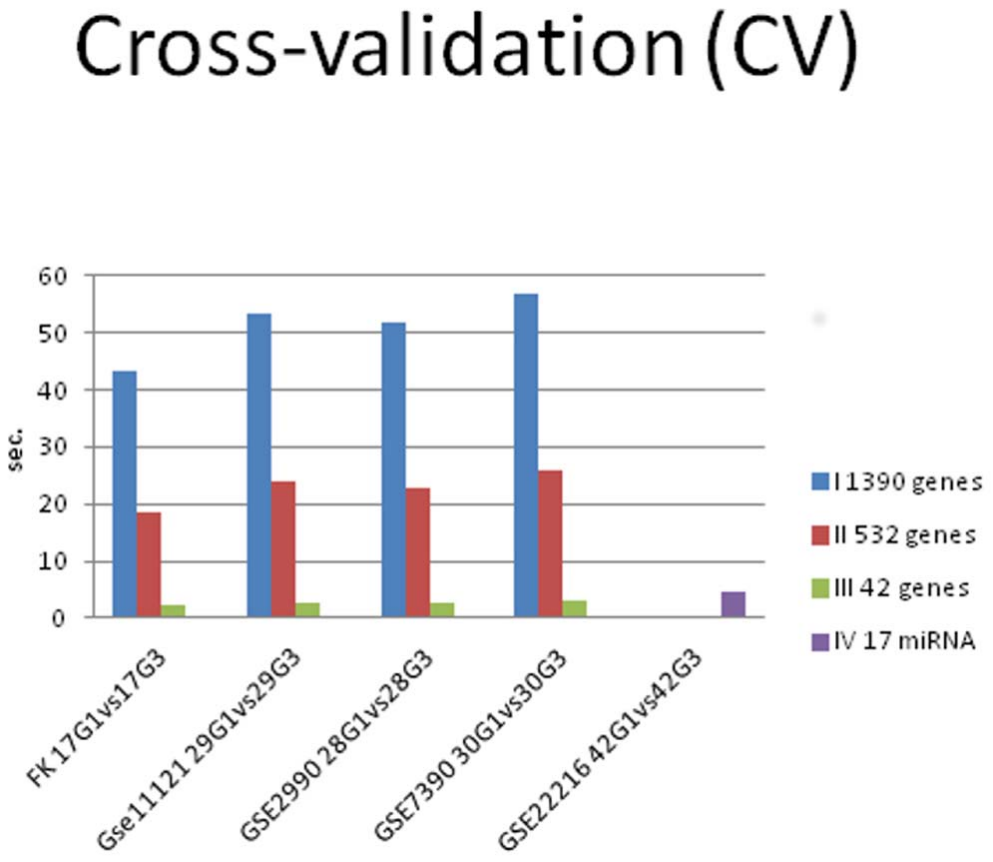
Bar chart for the time benchmark. The computational times required by the classification algorithm for the different proposed approaches with cross-validation (CV) were showed. (I: gene expression, II: combination of gene expression, and genome CNA, III: combination of gene expression - genome CNA, and miRNA analysis IV: miRNA classification)

**Figure 8:**
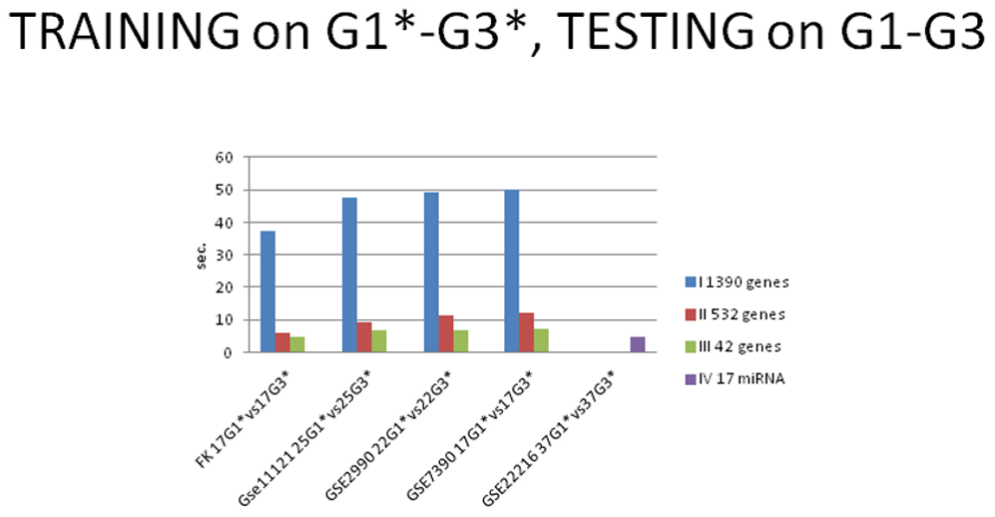
Bar chart for the time benchmark. The computational times required by the classification algorithm for the different proposed approaches with training on G1*–G3*, testing on G1–G3 (TT) were showed. (I: gene expression, II: combination of gene expression, and genome CNA, III: combination of gene expression - genome CNA, and miRNA analysis IV: miRNA classification)

Figure 7 shows bar chart for the computational time required by the classification algorithm for the different proposed approaches (I,II,III,IV) with cross-validation. Figure 8 shows bar chart for the computational time required by the classification algorithm for the different proposed approaches (I,II,III,IV) with training on G1*–G3*, testing on G1–G3 (TT).

The results on the evaluation of the combination approaches confirm the high potential of our method based on the combined effect of epigenetic and genetic changes resulting in deregulated gene expression and function in selecting genetic and epigenetic signatures suitable for BC grade classification, also for histological G2 BC re-classification in G1-like and G3-like BC tumors.

Our genetic (42-genes from approach III) and epigenetic signatures (17 miRNA from method IV) were able to correctly re-classify misclassified G2 BC tumors with no drop in performances with respect to signatures of genes identify by single differential gene expression analysis.

### Evaluation of our gene signatures in comparison with other gene signatures

Based on the comparison with Sotiriou et al. 97-gene signature, Ivshina et al. 18-gene signature and Ivshina et al. 6-gene signature, a down-sized gene signature (V approach) was obtained from our 42-gene signature, including only genes in common with the above considered gene signatures. A 4-based gene signature consisting of FOXM1, KPNA4, H2AFV and DDX19A was found. All the four genes were included in the Sotiriou et al. 97-gene signature, while only FOXM1 was comprised within the Ivshina et al. 18-gene signature. No genes were shared with the Ivshina et al. 6-gene signature.

### Evaluation of G1 and G3 classification

For each considered gene signature (our 42-gene signature, our 4-gene signature, Sotiriou et al. 97-gene signature, Ivshina et al. 18-gene signature and Ivshina et al. 6-gene signature, the results of G1 vs G3 BC classification are presented in Tables 11–14 for accuracy, sensitivity, specificity and AUC, respectively.

**Table 11:** G1 vs G3 classification performances (mRNA): accuracy, cross-validation.

ACCURACY					
	III 42 genes	V 4 genes	Sotiriou et al. 97 genes	Ivshina et al. 18 genes	Ivshina et al. 6 genes
GSE7390 30G1vs30G3	90.00% [CI 95%] 87.20–92.79	88.33% [CI 95%] 85.63–91.02	88.33% [CI 95%] 85.03–91.62	90.00% [CI 95%] 87.2–92.79	88.33% [CI 95%] 85.63–91.02
Stockholm 28G1vs28G3	90.00%[CI 95%] 86.50–93.49	90.00% [CI 95%] 86.50–93.49	90.00% [CI 95%] 87.10–92.89	91.33% [CI 95%] 88.32–94.33	88.00% [CI 95%] 84.03–91.96
**Mean (over datasets)**	**90%+/−0** 1	**89.16%+/−1.18** 1	**89.16%+/−1.18** 1	**90.66%+/−0.94** 1	**88.16%+/−0.23** 1

1 standard deviation.

**Table 12:** G1 vs. G3 classification performances (mRNA): sensitivity, cross-validation.

SENSITIVITY					
	III 42 genes	V 4 genes	Sotiriou et al. 97 genes	Ivshina et al. 18 genes	Ivshina et al. 6 genes
GSE7390 30G1vs30G3	100% [CI 95%] 100–100	90.00% [CI 95%] 86.13–93.86	93.33% [CI 95%] 89.95–96.7	93.33% [CI 95%] 89.95–96.7	83.33% [CI 95%] 79.11–87.54
Stockholm 28G1vs28G3	90.00% [CI 95%] 85.99–94.00	90.83% [CI 95%] 87.11–94.54	87.50% [CI 95%] 83.44–91.55	94.17% [CI 95%] 91.07–97.26	80.00% [CI 95%] 73.29–86.70
**Mean (over datasets)**	**95%+/−7-07** 1	**90.41%+/−0.58** 1	**90.41+/−4.12** 1	**93.75%+/−0.59** 1	**81.66%+/−2.35** 1

1 standard deviation.

**Table 13:** G1 vs G3 classification performances (mRNA): specificity, cross-validation.

SPECIFICITY					
	III 42 genes	V 4 genes	Sotiriou et al. 97 genes	Ivshina et al. 18 genes	Ivshina et al. 6 genes
GSE7390 30G1vs30G3	80.00% [CI 95%] 74.4–85.59	86.67% [CI 95%] 82.53–90.80	83.33% [CI 95%] 77.67–88.98	86.67% [CI 95%] 82.53–90.80	93.33% [CI 95%] 89.95–96.70
Stockholm 28G1vs28G3	90.00% [CI 95%] 84.41–95.58	90.00% [CI 95%] 85.99–94.00	93.33% [CI 95%] 89.83–96.82	88.33% [CI 95%] 83.52–93.13	96.67% [CI 95%] 94.05–99.28
**Mean (over datasets)**	**85%+/−7.07** 1	**88.33%+/−2.35** 1	**88.33%+/−7.07** 1	**87.5%+/−1.17** 1	**95%+/−2.26** 1

1 standard deviation.

**Table 14:** G1 vs G3 classification performance (mRNA): AUC, cross-validation.

AUC					
	III 42 genes	V 4 genes	Sotiriou et al. 97 genes	Ivshina et al. 18 genes	Ivshina et al. 6 genes
GSE7390 30G1vs30G3	0.900 [CI 95%] 0.86–0.93	0.900 [CI 95%] 0.86–0.93	0.911 [CI 95%] 0.88–0.93	0.878 [CI 95%] 0.83–0.91	0.922 [CI 95%] 0.89–0.95
Stockholm 28G1vs28G3	0.922[CI 95%] 0.88–0.96	0.911 [CI 95%] 0.87–0.94	0.864 [CI 95%] 0.82–0.90	0.943 [CI 95%] 0.91–0.96	0.894 [CI 95%] 0.84–0.93
**Mean (over datasets)**	**0.91+/−0.01** 1	**0.90+/−0.007** 1	**0.88+/−0.03** 1	**0.91+/−0.04** 1	**0.90+/−0.01** 1

1 standard deviation.

All signatures achieved good and similar mean performances. Slightly worsen mean sensitivity (81.66%) was found for the Ivshina et al. 6-gene signature but it outperformed in specificity. Similarly, slightly worsen mean specificity (85.00%) was found for our 42-gene signature but it outperformed in sensitivity.

### Evaluation of G2 classification

Accuracy, sensitivity, specificity and AUC, relative to the performance of the classifier for G1 vs G3 classification of BC patients employing the re-classified G2 patients (in G1* and G3*), for the above mentioned gene signatures are presented in Tables 15–18.

**Table 15:** Classification performances (mRNA): accuracy, TRAINING on G1*–G3*, TESTING on G1–G3.

ACCURACY					
	III 42 genes	V 4 genes	Sotiriou et al. 97 genes	Ivshina et al. 18 genes	Ivshina et al. 6 genes
GSE7390 17G1*vs17G3*	85%	88.33%	83.33%	85%	85%
Stockholm 19G1*vs19G3*	76.78%	82.14%	85.71%	89.28%	87.50%
**Mean (over datasets)**	**80.89%+/−5.81** 1	**85.23%+/−4.37** 1	**84.52%+/−1.68** 1	**87.14%+/−3.02** 1	**86.25%+/−1.76** 1

1 standard deviation.

**Table 16:** Classification performances (mRNA): sensitivity,TRAINING on G1*–G3*, TESTING on G1–G3.

SENSITIVITY					
	III 42 genes	V 4 genes	Sotiriou et al. 97 genes	Ivshina et al. 18 genes	Ivshina et al. 6 genes
GSE7390 17G1*vs17G3*	93.33%	96.66%	90%	93.33%	90%
Stockholm 19G1*vs19G3*	75%	75%	82.14%	89.28%	82.14%
**Mean (over datasets)**	**84.16%+/−12.96** 1	**85.83%+/−15.31** 1	**86.07%+/−5.55** 1	**91.30%+/−2.86** 1	**86.07%+/−5.55** 1

1 standard deviation.

**Table 17:** Classification performances (mRNA): specificity, TRAINING on G1*–G3*, TESTING on G1–G3.

SPECIFICITY					
	III 42 genes	V 4 genes	Sotiriou et al. 97 genes	Ivshina et al. 18 genes	Ivshina et al. 6 genes
GSE7390 17G1*vs17G3*	76.66%	80%	76.66%	76.66%	80%
Stockholm 19G1*vs19G3*	78.57%	89.28%	89.28%	89.28%	92.28%
**Mean (over datasets)**	**77.61%+/−1.35** 1	**84.64%+/−6.56** 1	**82.97%+/−8.92** 1	**82.97%+/−8.92** 1	**86.14%+/−8.68** 1

1 standard deviation.

**Table 18:** Classification performance (mRNA): AUC, TRAINING on G1*-G3P, TESTING on G1–G3.

AUC					
	III 42 genes	V 4 genes	Sotiriou et al. 97 genes	Ivshina et al.18 genes	Ivshina et al. 6 genes
GSE7390 17G1*vs17G3*	0.89	0.92	0.90	0.92	0.93
Stockholm19G1*vs19G3*	0.88	0.86	0.89	0.93	0.89
**Mean (over datasets)**	**0.88+/−0.007** 1	**0.89+/−0.04** 1	**0.89+/−0.007** 1	**0.92+/−0.007** 1	**0.91+/−0.02** 1

1 standard deviation.

All signatures achieved good and similar mean performances. Slightly worsen mean performance was found for our 42-gene signature. Best performance is achieved by the Ivshina et al. 18-gene signature (3 over 4 indexes). The Ivshina et al. 6-gene signature and our 4-gene signatures have very similar performance.

**Figure 9:**
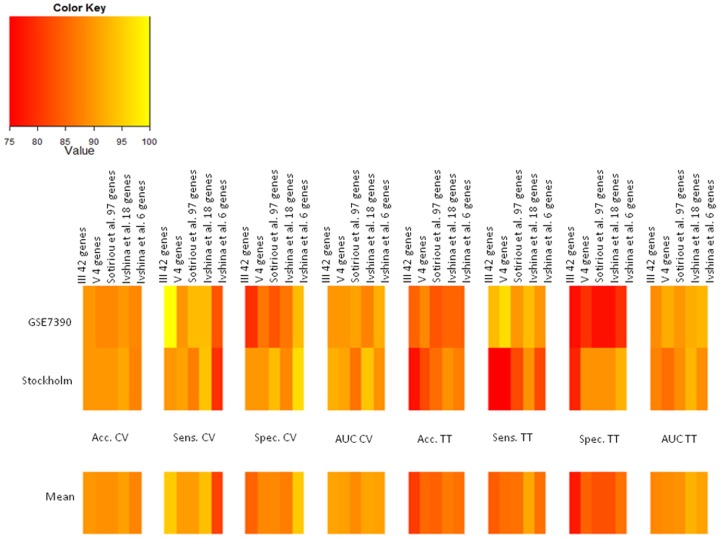
Heat maps for evaluation of our gene signatures in comparison with other gene signatures. III: combination of gene expression - genome CNA, and miRNA analysis, V: a down-sized gene signature (4 genes), Sotiriou et al. 97-gene signature, Ivshina et al. 18-gene signature and Ivshina et al. 6-gene signature. Classification performances were showed for: cross-validation (CV), and training on G1*–G3* - testing on G1–G3 (TT).

Figure 9 shows heat maps for evaluation of our gene signatures in comparison with other gene signatures (III, V,Sotiriou et al. 97-gene signature, Ivshina et al. 18-gene signature and Ivshina et al. 6-gene signature).

Results of Log-Rank tests for the comparison between G1*and G3*, G1 and G1*, G3 and G3* as classified by means of the abovementioned gene signatures, are shown in Table 19, 20, 21, respectively.

**Table 19:** Log-Rank Test G1*vs G3*.

LOG-RANK TEST (P-VALUE) G1*vs G3*					
	III 42 genes	V 4 genes	Sotiriou et al. 97 genes	Ivshina et al. 18 genes	Ivshina et al. 6 genes
GSE7390 17G1*vs17G3*	0.033	0.0003	0.0001	0.002	0.019
Stockholm19G1*vs19G3*	0.033	0.003	0.022	0.452	0.112

**Table 20:** Log-Rank Test G1*vs G1.

LOG-RANK TEST (P-VALUE) G1*vs G1					
	III 42 genes	V 4 genes	Sotiriou et al. 97 genes	Ivshina et al. 18 genes	Ivshina et al. 6 genes
GSE7390 17G1*vs17G1	0.652	0.773	0.680	0.609	0.393
Stockholm19G1*vs28G1	0.538	0.784	0.563	0.087	0.237

**Table 21:** Log-Rank Test G3*vs G3.

LOG-RANK TEST (P-VALUE) G3*vs G3					
	III 42 genes	V 4 genes	Sotiriou et al. 97 genes	Ivshina et al. 18 genes	Ivshina et al. 6 genes
GSE7390 17G3*vs17G3	0.308	0.963	0.679	0.854	0.651
Stockholm 19G3*vs28G3	0.414	0.373	0.322	0.876	0.890

Results of HR [C.I. 95%] for the comparison between G1*and G3*, G1 and G1*, G3 and G3* are shown in Table 22, 23, 24, respectively.

**Table 22:** Hazard ratio G1*vs G3*.

HAZARD-RATIO G1*vs G3*					
	III 42 genes	V 4 genes	Sotiriou et al. 97 genes	Ivshina et al. 18 genes	Ivshina et al. 6 genes
GSE7390 17G1*vs17G3*	2.801 [CI 95%] 1.048–7.488 p-value = 0.040	6.208 [CI 95%] 2.021–19.080 p-value = 0.001	8.920 [CI 95%] 2.423–32.840 p-value = 0.0009	3.872 [CI 95%] 1.520–9.860 p-value = 0.004	2.993 [CI 95%] 1.144–7.834 p-value = 0.025
Stockholm 19G1*vs19G3*	4.630 [CI 95%] 0.982–21.84 p-value = 0.052	11.669 [CI 95%] 1.475–92.310 p-value = 0.019	5.135 [CI 95%] 1.082–24.370 p-value = 0.03	1.549 [CI 95%] 0.491–4.885 p-value = 0.455	2.299 [CI 95%] 0.797–6.632 p-value = 0.123

**Table 23:** Hazard ratio G1*vs G1.

HAZARD-RATIO G1*vs G1					
	III 42 genes	V 4 genes	Sotiriou et al. 97 genes	Ivshina et al. 18 genes	Ivshina et al. 6 genes
GSE7390 17G1*vs17G1	1.390 [CI 95%] 0.330–5.855 p-value = 0.653	1.245 [CI 95%] 0.278–5.570 p-value = 0.774	1.370 [CI 95%] 0.303–6.178 p-value = 0.682	0.689 [CI 95%] 0.164–2.891 p-value = 0.611	0.541 [CI 95%] 0.129–2.266 p-value = 0.400
Stockholm 19G1*vs28G1	1.833 [CI 95%] 0.257–13.04 p-value = 0.538	1.397 [CI 95%] 0.126–15.41 p-value = 0.785	0.565 [CI 95%] 0.079–4.023 p-value = 0.569	0.264 [CI 95%] 0.051–1.363 p-value = 0.112	0.393 [CI 95%] 0.079–1.952 p-value = 0.254

**Table 24:** Hazard ratio G3*vs G3.

HAZARD-RATIO G3*vs G3					
	III 42 genes	V 4 genes	Sotiriou et al. 97 genes	Ivshina et al. 18 genes	Ivshina et al. 6 genes
GSE7390 17G3*vs17G3	0.654 [CI 95%] 0.287–1.489 p-value = 0.312	1.018 [CI 95%] 0.472–2.195 p-value = 0.963	1.181 [CI 95%] 0.533–2.617 p-value = 0.680	1.075 [CI 95%] 0.494–2.341 p-value = 0.855	1.204 [CI 95%] 0.537–2.698 p-value = 0.652
Stockholm 19G3*vs28G3	1.483 [CI 95%] 0.571–3.848 p-value = 0.418	0.660 [CI 95%] 0.261–1.667 p-value = 0.380	0.616 [CI 95%] 0.236–1.606 p-value = 0.322	0.924 [CI 95%] 0.344–2.486 p-value = 0.877	0.935 [CI 95%] 0.360–2.427 p-value = 0.890

**Figure 10:**
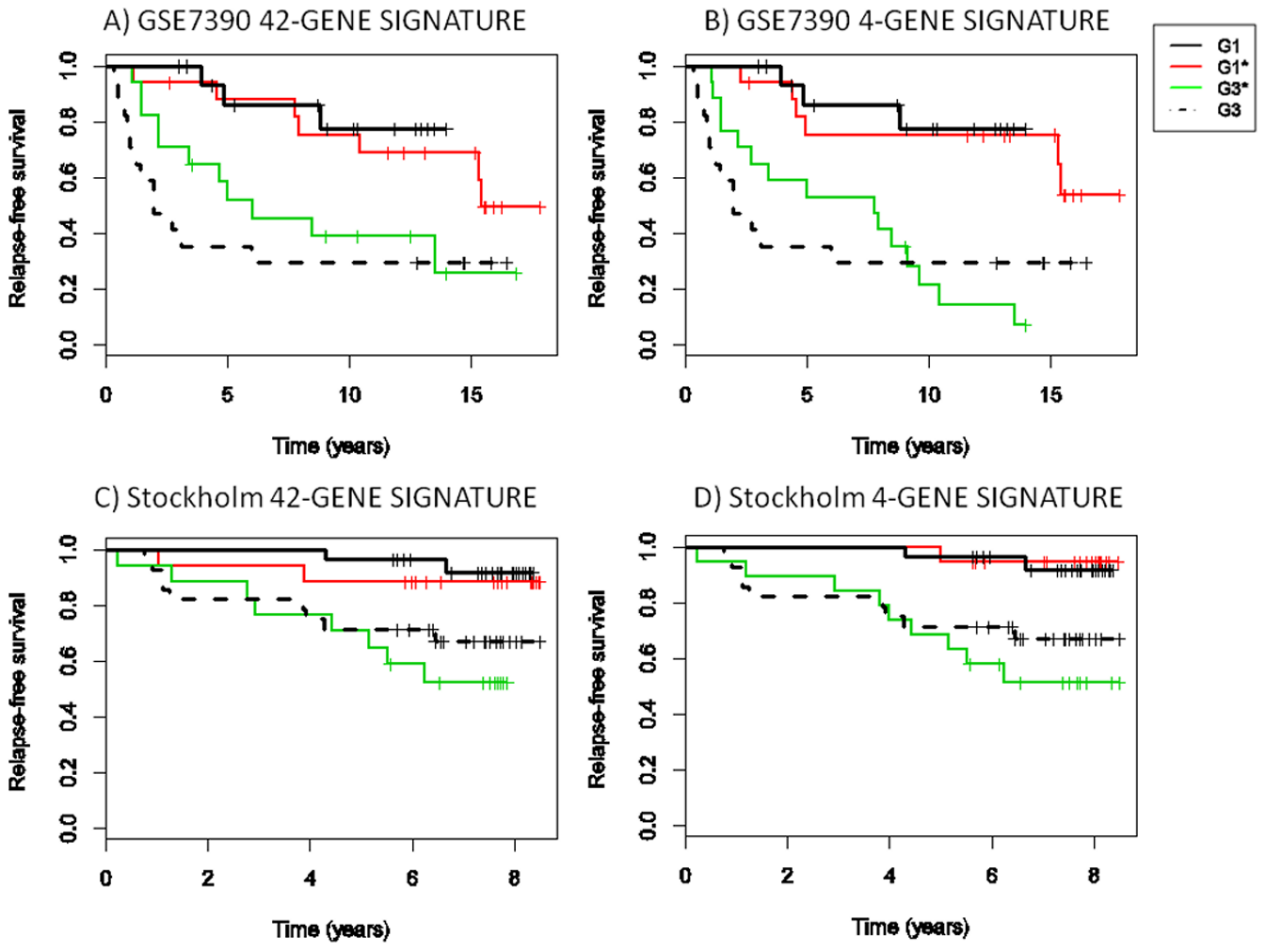
Relapse-free survival analysis for GSE7390 and Stockholm datasets. Kaplan-Meier survival curves for G3* (green) and G1* (red) superimposed on survival curves of histologic G1 (black line) and histologic G3 (dotted line) A) GSE7390 42-gene signature; B) GSE7390 4-gene signature; C) Stockholm 42-gene signature; D) Stockholm 4-gene signature.

Relapse-free survival curves of G1* vs. G3* patients, as re-classified from histological G2 by the use of our 42-gene signatures and 4-gene signatures, are shown as representative examples, in figure 10 for GSE7390 (a–b) and Stockholm (c–d) datasets, respectively.

GSE7390 patients re-classified as G1* showed significantly less risk of recurrence than those re-classified in G3* using both our 42-gene and 4-gene signatures (42-gene signature: log-rank test p = 0.033, HR  =  2.801 [C.I 95%] 1.048–7.488 p = 0.040; 4-gene signature: log-rank test p = 0.000321, HR  = 6.208 [C.I 95%] 2.021–19.08 p = 0.00143).

No significant difference was observed between the G1* and G1 (42-gene signature: log-rank test p = 0.652, HR  = 1.390 [C.I 95%] 0.330–5.855 p = 0.653; 4-gene signature: log-rank test p = 0.773, HR  = 1.245 [C.I 95%] 0.278–5.570 p = 0.774) and G3* and G3 (42-gene signature: log-rank test p = 0.308, HR  = 0.654 [C.I 95%] 0.287–1.489 p = 0.312; 4-gene signature log-rank test: p = 0.963, HR  = 1.018 [C.I 95%] 0.472–2.1954 p = 0.963).

Similarly, Stockholm patients re-classified as G1* showed significantly less risk of recurrence than those re-classified in G3* using both our 42-gene and 4-gene signatures (42-gene signature: log-rank test p = 0.0331, HR  = 4.63 [C.I 95%] 0.982–21.84 p = 0.05; 4-gene signature: log-rank test p = 0.00316, 11.669 [C.I 95%] 1.475–92.31 p = 0.0199).

No significant difference was observed between the G1* and G1 (42-gene signature: log-rank test p = 0.538, HR  = 1.833 [C.I 95%] 0.257–13.04 p = 0.538; 4-gene signature: log-rank test p = 0.784, HR  = 1.397 [C.I 95%] 0.126–15.410 p = 0.785) and G3* and G3 (42-gene signature: log-rank test p = 0.414, HR  = 1.483 [C.I 95%] 0.571–3.848 p = 0.418; 4-gene signature log-rank test: p = 0.373, HR  = 0.660 [C.I 95%] 0.261–1.667 p = 0.380).

Our 42-gene signature and 4-gene signature were found able to perform G2 re-classification with similar, in some cases, better, prognostic value than the other gene signatures. Specifically: 1) our 42-gene signature and 4-gene signature have better performance than Ivshina et al. signatures (both 18-genes and 6-genes) in the log-rank test and in the HR test when a statistical threshold of 0.05 is set (see results for Stockholm dataset); 2) our 4-gene signature has better performance in log-rank test than both Sotiriou et al. signature and Ivshina et al. signature (both 18-genes and 6-genes) when a statistical threshold of 0.01 is set (see Stockholm results).

## Discussion

In the management of BC, the identification of subgroups of patients with different prognoses and responses to treatment [72] [95] is relevant for therapeutic planning. Recently, classification of BC based on gene expression profiling has been proposed [12] [96]–[97]. Furthermore, some authors developed gene expression signatures which are capable of discerning BC tumors of G1 and G3 histology, providing a more objective measure of grade with prognostic benefit for patients with G2 disease [14]–[15]. However, these gene signatures have few genes in common notwithstanding they are derived from similar approaches. Therefore, results stemming from alternative, different approaches may help in validating or enriching these signatures.

Few studies have examined classification methods based on a combination of different genome-wide microarray-based expression profiling approaches. New models of oncogenomic progression should examine the combined consequence of epigenetic (miRNAs) and genetic (CNA) changes as concomitant causation of tumor heterogeneity. Previous studies have indicated how such genetic and epigenetic changes can influence gene expression, and thus tumor evolution [98]. It is less clear how these mechanisms influence each other and how these cumulative changes co-evolve and influence gene expression during tumorigenesis [98].

In this paper, we showed that a classification analysis relevant for disease progression in BC as characterized by grade definition can be based on different combination approaches under genetic and epigenetic interaction assumptions (I: gene expression, II: combination of gene expression and genome CNA, III: combination of gene expression, genome CNA and miRNA analysis, IV: miRNAs, as obtained from the combined analysis of gene expression and genome CNA). Our results showed that integration of these various genetic data is effective for BC classification of G1 and G3 samples but also of G2 samples, this approach resulted to ameliorate cancer classification.

Although the purpose of our study is similar to that of previous investigations based on traditional differential gene expression analysis [14]–[15], some novel and incremental aspects need to be acknowledged:

Our method to select the gene signatures (based on the combination approach) has been implemented for the first time, to our knowledge, for improving grade definition in BC, in particular for the re-classification of histological G2 BC into G1-like and G3-like tumors; this could allow new cross-validation studies with different methods for the in silico validation of biomarkers. Furthermore, our proposed methodology could be very useful for understanding the interactions between mRNA, CNA, and miRNA, and further studies should be conducted to these purposes.
Epigenetic signatures able to perform grade classification as those obtained by method IV have been identified and validated for the first time to our knowledge: this could open new investigations on the role of miRNA e.g. circulating in blood. For instance, recent evidence points to small non-coding miRNAs as promising biomarkers for the detection of several human tumors [96], given their strong stability against RNase digestion. This evidence, added with the fact that miRNAs detection by quantitative polymerase chain reaction (qPCR) is sensitive and robust, make miRNAs potentially important tools for cancer diagnosis. Moreover, plasma miRNAs are potentially able to monitor asymptomatic high-risk individuals, in order to detect early stage BC and discriminate between benign and malignant disease (see [66] for a review). Moreover, the translation of miRNAs signature into clinical assays seems to be more feasible and less expensive: for instance, the application of this miRNA profile screening on small specimens, as fine needle aspiration, core biopsy material, or small aliquots of blood, seems to be more suitable and a viable cost-effective alternative to more expensive commercial products for the immunohistochemichal profile techniques usually applied to a standard pathology block.
A 42-gene signature and a 4-gene signature have been developed, the latter obtained through a meta-analytical approach: these gene signatures resulted to perform grade classification and G2 re-classification with performances similar to other gene signatures previously published and with similar or even better prognostic value in some cases.
The number of genes in the downsized gene signature (4 gene) is significantly lower than published signatures facilitating the implementation of a clinic assay (Sotiriou et al. 97-gene signature, Mammaprint 70-gene signature [12], Oncotype 21-gene signature [13], Ivshina et al. 18-gene signature, Ivshina et al. 6-gene signature). The 42- gene signature has instead a number of genes, which allow experimental validation in laboratory with limited costs. Similar considerations are valid for the 4 miRNA (derived from the 4-gene signature) and the 17 miRNA (derived from the 42-gene signature).

Our classification algorithm was build on 42 genes. These were obtained by the above described combination approach. Other classification methods have been proposed and used, based on a limited number of genes, obtained by different approaches. In Sotiriou et al. the standardized mean difference of Hedges and Olkin [99] was used to rank genes by their differential expression. They used the max T algorithm of Westfall and Young [100] to correct for multiple testing with an extension proposed by Korn et al. [101] to control the number of false discoveries, taking into account the dependencies between genes. This strategies allowed them to obtain 97 genes. Ivshina et al. ran the PAM algorithm [102] with all probe sets as input, and acquired a minimal set of probe sets which gave: 1) the lowest misclassification (error) rate, and 2) a secondary minimum on the error curve. This strategy allowed them to obtain 18 and 6 genes, respectively. As shown in our results (table 11–18) all classification methods achieved very good results. However, as reported in several studies [103]–[105], SVM and predictive methods have some limitations, since they could lead to too optimistic statement. Meyer et al. [103] compared SVM to several other classification and regression methods, by means of standard performance measures. This comparison showed that all predictive methods have good performances, and SVM did not demonstrate its overall superiority. Parikesit et al. [104] and Smith et al. [105] assessed different predictive methods applied to gene annotations, and reported that predictions should be chosen carefully in order to avoid introducing biases.

Although validation is necessary in independent wet lab experiments, the new genomic features identified in our work shed new light on the biology underlying histological grade in breast cancer, as evidenced in the following section.

### Phase I: Genome profile analysis of G1 vs G3 genetic profile

In our study, we found that BC of histologic G1 and G3 exhibits a distinct expression profile with altered expression of 1190 genes (578 up and 612 down) involved in cell proliferation, in particular in cell cycle control and mitosis, and in DNA repair and stability. Many of our up-regulated G3 genes overlap with those found by Sotiriou et al. [14], (75 over 80, 82.5%) and with those found by Ivshina A. et al. [15], (10 over 16,62.5%). Among our down-regulated G3 genes we found 11 over 19 indicated (57.9%) by Sotirou et al. [14].

Namely, FOXM1, MYBL2 and TPX2 are genes found in our lists of up-regulated G3 genes as well as in the lists of Sotiriou and Ivshina, revealing that the increase of these genes, regulating the DNA transcription and controlling the spindle formation during mitosis, could be important for the development of G3 tumor.

### Phase I: Genome profile analysis of G2

When we compared our G2 genetic profile with G1 and G3 mRNA expression, we found that all G2 altered genes belong to either G3 up-regulated (G1 downregulated) or to G3 downregulated (G1 upregulated) classes, confirming previous results described in Sotirou et al. and by Ivshina A. et al. [14]–[15].

### Phase II analysis: CNA Integration analysis

The Phase II analysis of CNA revealed that there are specific areas affected by deletion or amplification in BC G1 and G3. The affected regions comprise chromosome 3p13, 8q21-24 and 8q22.

The deletion of chromosome 3p13 has been observed frequently in epithelial cancers of several organs [106] and in prostate cancer [107]. The 3p13-21 region, in particular, encodes for the Rho-like GTPase gene ARHGEF3, which, as a guanine nucleotide exchange factor for Rho family members, could have a role in oncogenic transformation [108].

The copy number gain of genes in chromosomal region 8q21-24 has been demonstrated to be associated with genesis and progression of prostate cancer (PCa) [109] with a significant amplification of E2F5 and MYC genes, the former being included among our G3 up/amplified genes.

Moreover, in BC, the amplification of 8q22 region leads to the over-expression of YWHAZ gene (tyrosine 3-monooxygenase/tryptophan 5-monooxygenase activation protein, zeta polypeptide), a typical feature of BC resistant to anthracycline treatment [110]–[111].

### Phase III and IV analysis: miRNA and mRNA target analysis

The Phase III and IV analysis revealed that there are profiles of target mRNA (42-genes) and of their miRNAs (17 miRNAs) showing promising results regarding BC grade definition.

As the used dataset identified by phase III contains differentially expressed miRNAs between G1 and G3, in order to obtain an expression profile of the miRNAs that describe each grade, and eventually identify prognostic genes and miRNAs expression signatures, we compared the up- and down-regulated miRNAs and their target mRNA in G1 vs G3. We obtained (phase IV) a set of miRNAs that were up-regulated in G3 (class 1), and thus down-regulated in G1 (class 2), as well as a set of miRNAs down-regulated in G3 (class 3), and thus up-regulated in G1 (class 4). Detailed analysis of the miRNAs in each class revealed common miRNAs among classes 1 and 2. The same was observed for miRNAs of classes 3 and 4. Fig. 5 shows a list of the miRNAs of each grade identified in our study in relation to the most relevant putative targets.

### miRNA analysis and target identification/description: class 1 and class 2

Class 1 is characterized by the up-regulation of several miRNAs: *Hsa-miR-532-5p, Hsa-miR-188-5p, Hsa-miR-515-5p, Hsa-miR-362-5p, Hsa-miR-142-3p, Hsa-miR-941,* and *Hsa-miR-455-5p* (Fig. 5). Although the role of Hsa-miR-532-5p expression in BC remains to be clarified, it has been shown to be significantly up-regulated in melanoma lines and metastatic melanoma tumours relative to normal melanocytes and primary melanomas, respectively [112]. Among its possible targets, we identified EF3, which, being a Rho-like GTPase, has an important role in many cellular processes (cytoskeletal rearrangements, transcriptional activation, regulation of cell morphology and cell aggregation, cytokinesis, endocytosis and secretion), but also is possibly involved in oncogenic transformation [113]. This gene is a possible target of Hsa-miR-362-5p, a miRNA of the melanoma signature [114], and Hsa-miR-515-5p, already involved in BC development [115]. Hsa-miR-532-5p regulates also interleukin 6 signal transducer (IL6ST), a signal transducer that could be important for interleukin 6 inflammatory role in BC [116]–[117]. This gene could be a target also of Hsa-miR-188-5p, already described in rectal cancer [118]. Hsa-miR-532-5p regulates also stanniocalcin 2 (STC2): it is a glycoprotein hormone that plays an important role in calcium and phosphate homeostasis and is considered a tumor progression predictor for gastric cancer [119] and breast carcinoma [120]. Concerning Hsa-miR-188-5p, it already results to be overexpressed in UVB irradiated mouse skin [121], suggesting a potential role of this miR in response to oxidative stress linked to radiation. As oxidative stress response is one of the active pathways of tumor cells (see [122] for a review), thus this miRNA is expected to be up-regulated in G3 tumor samples. Among its possible target genes, clusterin (CLU) is a pro-proliferative gene and one of the genes induced by exposure to ionizing radiation (IR) [123], which usually causes oxidative damage, while neurobeachin (NBEA), a lysosomal-trafficking regulator, is one of the genes of the common fragile site regions [124]. Class 1 comprises also Hsa-miR-515-5p, that is usually down-regulated by estrogen receptor in BC [125]. In our case this miRNA in unexpectedly up-regulated; this may possibly due to the fact that our database contains both ER+ and ER- BC samples. Hsa-miR-515-5p regulates a growth factor signalling transducer, the ring finger protein-like 1 (ZFP36L1), which has a role in growth control of BC cell line [126]. In class 1 we found also Hsa-miR-142-3p, that, with Hsa-miR-532-5p, is a circulating miRNA already considered biomarker of colorectal carcinoma [127]. Two possible targets of this miRNAs are fibronectin type III domain containing 3A (FNDC3A), whose expression controls cell adhesion, migration and proliferation [128] and myotubularin related protein 9 (MTM9), whose chromosomal gain is considered a prognostic event in oesophageal adenocarcinoma [129]. The last gene is also a possible target of Hsa-miR-941, that is a circulating biomarker of ulcerative colitis [130]. Hsa-miR-455-5p has already been found to be up-regulated in different types of tumors, as basal cell carcinoma of the skin [131], endometrial adenocarcinomas [132], but it also has a diagnostic value in laryngeal cancer [133] and in hepatocellular adenoma [134]. Its target tight junction protein 1 (TJP1), being a protein of the cytoplasmic membrane surface of intracellular tight junction, could have a role in communication among two cells by cell-cell junction. A role for TJP1 in controlling epithelial cell integrity in BC cells has been pointed out [135].

It is not a coincidence to find in G3 BC several upregulated miRNAs common to melanoma (Hsa-miR-532-5p, Hsa-miR-362-5p and Hsa-miR-455-5p), as Axelsen JB et al. [136] reported that the genes selectively altered in BC majorly overlap with the ones altered in melanoma.

In contrast, in class 2 we observed the up-regulation of 3 microRNAs, as depicted in Fig. 5. These miRNAs regulate several genes, two of which could be considered transcription modulators (aminoadipic semialdehyde synthase, GON4L and zinc finger protein, FOG family member 2, ZFPM2), and are able to control the transformation process of BC cells [137]. The up-regulated miRs are *Hsa-miR-532-5p*, already described in BC [138], *Hsa-miR-455-5p* and *Hsa-miR-515-5p*. Hsa-miR-532-5p regulates lipoma preferred partner (LPP), an indispensable regulator of migration [139], and the aminoadipate-semialdehyde synthase (AASS) gene, involved in lysine degradation pathway. No role in cancer progression for the last gene has been published yet. Hsa-miR-455-5p has been already correlated with vascular invasion of endometrial serous adenocarcinomas [132]. The main target of this miRNA is calcium-dependent secretion activator 2 (CADPS2), a protein that facilitates the secretion and trafficking of dense-core vesicles [140], a process necessary for tumoral cell communication. The last miRNA is Hsa-miR-515-5p; the down-regulation of this miRNA has been found in ER-positive BC associated with cell proliferation [141] and controls possibly a zinc-finger proteins that regulates the expression of GATA-target genes, thus modulating mammary gland differentiation or involution [137].

### microRNA analysis and target identification/description: class 3 and class 4

Class 3 is characterized by the down-regulation of 8 miRNAs, as shown in Fig. 5. *Hsa-miR-372* is already a potential marker of lung cancer [142], and regulates an ATPase family member ATAD2, a translocase of inner mitochondrial membrane 17 (TIMM17A) and the transcription factor E2F5. ATAD2 is within a commonly amplified region (8q24) across multiple cancer types [95] and its expression seems to be a predictor of poor prognosis in BC [143] and in other tumors, as in prostate cancer [144]–[145]. TIMM17A expression is associated with poor pathological and clinical outcome of BC [146]–[147]. E2F5 is cell cycle-related transcription factor overexpressed in ER-negative BC, [148] and is also considered a biomarker of worse clinical outcome [149]. This gene is also a possible target of *Hsa-let-7c*. Hsa-let-7c is a member of a tumor-suppressor microRNA family, often inactivated in human malignancies, in particular in BC [150]–[151] and in prostate cancer [152].

*Hsa-miR-320d*, already found down-regulated in colon cancer cells [153], and *Hsa-miR-139-5p,* regulate genes involved in proliferation control (antizyme inhibitor 1, AZIN1; forkhead box M1, FOXM1; RAD51 associated protein 1, RAD51AP1) [154]–[156], and transcription regulation (DNA replication and sister chromatid cohesion 1, DSCC1; RAD21 yeast homologue, RAD21) key processes for G3 development. Loss of *Hsa-miR-139-5p* have been reported in different tumor specimens of esophageal squamous cells [157], clear renal cell carcinoma [158] and BC samples [159], underscoring its potential role as biomarker for screening and early detection of these tumors. Among Hsa-miR-139-5p possible targets we found H2AFV, an histone family member, and Lysosome Transmembrane Protein 4-Beta (LAPTM4B), an oncoprotein originally identified in hepatocellular carcinomas [160]–[161], whose over-expression has been already associated with BC susceptibility and prognosis [162]–[163].

*Hsa-miR-125-5p* has been described as a tumor suppressor. Its down-regulation has been already associated with several types of cancer, such as BC [164]–[166], ovarian cancer [167], lung cancer [168], and medulloblastoma [169]. Moreover, its down-regulation has been found in the blood of BC patients [170]. Its possible target is a protein involved in the process of proper folding of secretory proteins, Sec61 alpha 2 subunit (SEC61A2). The correct assembly of newly synthesised secreted proteins is a key step for tumors to invade the surrounding microenvironment.

The last three miRNAs in class 3 are *Hsa-miR-567*, *Hsa-miR-647* and *Hsa-miR-328*. The first miRNA, already associated with colorectal cancer [171], regulates polymerase (RNA) II polypeptide K (POLR2K), which is involved in the transcription of DNA into RNA. This enzyme is also over-expressed in hepatocellular carcinoma [172]. The other three targets are S-phase kinase protein 2 (Skp2), a pro-proliferative, oncogenic protein overexpressed in human BC [173], transmembrane protein 70 (TMEM70), encoded by 8q21 region amplified in BC [174], and importin alpha 3 (KPNA4), a p53 stability regulator that can influence its transcription [175]. The second miRNA, Hsa-miR-647, already described as a possible prostate cancer recurrence predictor [176], could influence the level of transcription by modulating the Transcription Elongation Factor B (TCEB1), a target of Hsa-miR-320d. The last miRNA, Hsa-miR-328, responsible for the development of drug resistance in BC cell lines [177], is possibly able to regulate the already described YWHAZ gene.

Class 4 is characterized by several up-regulated G3 miRNAs. The possible relation among this microRNAs and cancer has been already described in class 3, as all of them, except *Hsa-miR-627 and Hsa-miR-581*, are down-regulated in G3. Several of these miRNAs (*Hsa-let-7c, Hsa-miR-372, Hsa-miR-139-p*) controls transcription regulators (ASF1B, already described; DEAD (Asp-Glu-Ala-As) Box Polypeptide 19A, DDX19A; H2AFV, already described; Hematological And Neurological Expressed 1 Protein, HN1). For some of the target genes, as for ASF1B, a role as a predictor of poor outcome in BC, when over-expressed, has already been described [178]; thus this gene is expected to be found potentially down-regulated in G1 BC. Some miRNAs regulate cell cycle and proliferation (*Hsa-miR-139-5p, and Hsa-miR-320d*) by repressing G protein-coupled receptor 56 (GPR56), whose over-expression plays an inhibitory role in melanoma progression [179], by controlling ribosome assembly (Nuclear Import 7 Homolog, NIP7) [180], and by regulating proliferation, repressing SHC SH2-domain binding protein 1 (SHCBP1), that is a proliferation controller downstream of Shc [181]. Two miRNAs (*Hsa-miR-372 and Hsa-miR-125a-5p*) regulate protein folding by repressing two chaperones (DNAJA2 and DNAJC9) and SEC61A2, already described as a fundamental complex for the correct protein folding. Specifically concerning *Hsa-miR-627* and *Hsa-miR-581*, which are the only miRNAs not in common between class 3 and 4, it has been proposed for both an unexpected role of inhibitor of proliferation of colon cancer cells [182], and hepatocellular carcinoma respectively [183]. Concerning their target, we found an anti-apoptotic factor (tripartite motif containing 39, TRIM39) [184] and NIP7, already described. For these two targets a direct role in BC development has not been described yet.

### The 4-gene signature

The down-sized 4-gene signature identified through our meta-analysis, consisting of FOXM1, KPNA4, H2AFV and DDX19A, represents a highly relevant finding for the biology underlying histological grades in BC, in particular regarding the cell proliferation, and DNA stability.

The forkhead box (Fox) M1 gene belongs to a superfamily of evolutionarily conserved transcriptional regulators that are involved in a wide range of biological processes, as the control of mammary gland differentiation [185] and cell proliferation [186], having a fundamental role in both G1/S phase progression [186], but also stimulating the expression of several critical genes of the G2/M phase (cyclin B, Aurora B, CDC25B, CENPA, Survivin) [187]. Its deregulation has been implicated in cancer growth, survival, and chemotherapy resistance [186]. For instance, in normal breast mammary gland, FOXM1 expression is often weak and only localized in proliferating cells [188]–[189], whilst it is over-expressed in various human malignancies (breast, prostate, lung, ovary, colon, pancreas, …) [190]. In BC the levels of FOXM1 correlate positively with the tumor grade [191] and with poor prognosis [189] [192]. A recent publication [193] suggests that FOXM1 promotes cancer invasion and metastasis via TGF-beta/SMAD3 pathway activation, but it is also able to induce EMT-like changes in hepatocellular carcinoma for a review see [194], demonstrating a clear role of this gene in G3 hystologic phenotype.

The other 3 genes of our 4-gene signature are different isoforms of genes found also in Sotiriou signature [14]: KPNA4 (karyopherin alpha 4 or importin alpha 3), H2AFV (H2A histone family, member V) and DDX19A (DEAD box polypeptide 19A).

KPNA4 belongs to the family of importins or karyopherins, which are responsible for the translocation of the cargo protein across the nuclear membrane. Several onco-proteins, such as BRCA1 (BC susceptibility gene 1), need to translocate across the nuclear membrane to reach the correct localization for their oncogenic function. One of the possible KNPA4 target is STAT3 protein, whose aberrant activation and translocation into the nucleus promotes initiation and progression of human cancers by either inhibiting apoptosis or inducing cell proliferation, angiogenesis, invasion, and metastasis [195]. This is probably the reason why G3 BC samples over-expressed KPNA4. Moreover, another member of the importins family, KPNA2, is uniformly up-regulated across cancer types and proposed as poor prognostic cancer marker [196]. KPNA2 has been found also by Sotirou et al.

H2AFV is one variant of the histonic component of the nucleosome. Its function is mainly to control the compactness of the chromatin and to recruit the transcriptional machinery for gene activation, thus regulating DNA transcription, replication and chromosomal stability. One of the histone isotypes, H2Ac, is already involved in estrogen receptor-positive clinical breast cancer tissues, where it regulates ER-target genes [197]. Another variant, H2A.Z, reported in several types of cancers, is causally linked to genomic instability and tumorigenesis [198]; it has been associated with lymph node metastasis and decreased BC survival [199]. It is not unexpected to find H2AFV in the class of up/amplified G3 genes.

The last 4-gene signature BC marker, DDX19A, is an ATP-dependent RNA helicase involved in mRNA export from the nucleus and in remodeling of ribonucleoprotein particles. In eukaryotic cells, gene expression is intimately tied to nuclear export of properly transcribed, processed, and assembled messenger ribonucleoprotein complexes (mRNPs). Post-transcriptional control of gene silencing by miRNAs is a ribonucleoprotein-driven process, which involves specific RNA binding proteins, miRNAs and their mRNA targets. This multi-component RNA-induced silencing complex (miRNA-RISC) regulates the stability and translation of mRNAs that are partially or fully complementary to specific miRNAs. It is possible that DDX19A has a role in the unwinding of microRNAs duplex during the miRNA maturation process, as the DEAD-protein, ATP-dependent RNA helicase p68 has a role in let-7 microRNA pathway maturation [198], in cancer cell proliferation and metastasis [200]–[201].

### miRNAs associated with the 4-gene signature

The analysis of the miRNA profile associated to our 4-gene signature allowed us to identify 4 miRNAs, that have the 4 genes of the signature as potential targets.

The miRNA able to control FOXM1 is Hsa-miR-320d, that has been already found down-regulated in colon cancer stem cells [153]: for instance, cancer stem cells isolated from the hepatocellular HT29 cell line showed down-regulation of Hsa-miR-320d, and it has been suggested that the down-regulation of this miRNA is important for carcinogenesis [153], while its expression is associated with the probability of recurrence-free survival in stage II colon cancer patients [202]. This miRNA is particularly significant because it has been already found in serum as a potential biomarker for detecting acute myeloid leukemia [203], making it an ideal, potential, circulating BC marker.

We have introduced in this paper the potential role of *Hsa-miR-139-5p* in the regulation of genes involved in proliferation control. Loss of Hsa-miR-139-5p has been reported in different tumor specimens of esophageal squamous cells [157], clear renal cell carcinoma [158] and BC samples [159], highlighting its potential role as a biomarker for screening and early detection of these tumors. Among Hsa-miR-139-5p possible targets, we found H2AFV, a histone family member, which is supposed to be upregulated in G3 and downregulated in G1. The dicotomic role of this miRNA has been already described in esophageal squamous cell carcinoma (ESCC) [204] [157], where reduced levels of this miRNA are associated with lymph node metastases, while its expression is found in adjacent non-cancerous tissue from ESCC patients, miming the G3 vs G1 situation. Moreover, Hsa-miR-139-5p expression seems to induce cell cycle arrest in G0/G1 phase and to suppress the invasive capability of the cells, and in MCF7 breast cancer cell line it has been proposed as a regulator of metastatic process [205]. Hsa-miR-139-5p has been found in circulating exosome of lung cancer patients plasma [68], making this miRNA another new possible biomarker for BC diagnostic application.

The gene importin alpha 3 (KPNA4), a p53 stability regulator that can influence its transcription [175], is one of the target of *Hsa-miR-567*. This miRNA is one of the colorectal cancer miRNA encoded by regions subjected to microsatellite sequence instability [171]. The role of this miRNA in BC development still needs to be unravelled.

DEAD (Asp-Glu-Ala-As) Box Polypeptide 19A (DDX19A) transcription factor is controlled by Hsa-let-7c. Hsa-let-7c is a member of a tumor-suppressor 10-microRNAs family [206], related often to human malignancies, in particular BC [150]–[151] and prostate cancer [152]. The *let-7* miRNAs have been shown to regulate multiple oncogenes such as HMGA2, c-Myc, RAS, and cyclinD1 [207]–[210]. Although in lung cancer [211]–[212] let-7c has been found as a tumor suppressor, from our analysis it resulted to be upregulated in G3 BC samples. This behaviour seems to resemble the one identified for prostate cancer, where the over-expression of Let-7c may be involved in the metastatic process, given that it is significantly associated to high grade prostate carcinoma [213].

## Conclusion

A combination of genetic and epigenetic changes has been shown to contribute to development of human cancer resulting in deregulation of gene expression and function. Genetic changes result in widespread deregulation of gene expression profiles and the disruption of signaling networks that control normal cell proliferation and functions. In addition to changes in DNA and chromosomes, oncogenic processes can be profoundly influenced by epigenetic mechanisms. While there has been considerable progress in understanding the influence of genetic and epigenetic mechanisms in tumorigenesis, few studies have examined the consequences of the interplay between these two processes. Before implementing integration methodologies, the interactions between mRNA, CNA, and microRNA should be investigated.

In this study, we integrated for the first time the analysis of mRNA expression, CNA, and miRNA expression for the definition and selection of limited genomic and epigenomic signatures useful for improving grade classification in human BC. From mRNA expression results, we found, consistently with previous published results, that grade 2 BC is most likely a mixture of misclassified grade 1 and grade 3, given that it can be accounted for by the gene signature of either grade 1 or grade 3. The combination of mRNA profile analysis and copy number data with microRNA expression levels led to identification of two gene signatures of 42 and 4 altered genes (FOXM1, KPNA4, H2AFV and DDX19A) respectively, the latter obtained as a result of a meta-analysis including previous relevant studies. The 42-based gene signature identifies 4 classes of up- or down-regulated microRNAs (17 microRNAs) their 17 target mRNA, while the 4-based genes signature identified 4 microRNAs (Hsa-miR-320d, Hsa-miR-139-5p, Hsa-miR-567 and let-7c).

Our identified mRNAs and microRNAs were validated as a classifier for BC grade and relatively to prognostic factors, and their limited number could potentially facilitate the implementation of assays for laboratory validation.
